# Obstructive Sleep Apnoea and Risk of Fragility Fracture in Patients With Type 2 Diabetes: A Population‐Based Retrospective Cohort Study

**DOI:** 10.1002/edm2.70100

**Published:** 2025-11-07

**Authors:** Esraa A. Makhdom, Anuradhaa Subramanian, Krishnarajah Nirantharakumar, Nicola J. Adderley, Abd A. Tahrani

**Affiliations:** ^1^ Department of Metabolism and Systems Science University of Birmingham Birmingham UK; ^2^ Department of Respiratory Care Imam Abdulrahman Bin Faisal University Dammam Saudi Arabia; ^3^ Centre for Endocrinology, Diabetes and Metabolism, Birmingham Health Partners Birmingham UK; ^4^ Department of Applied Health Sciences University of Birmingham, Birmingham Birmingham UK; ^5^ National Institute for Health and Care Research (NIHR) Birmingham Biomedical Research Centre Birmingham UK; ^6^ University Hospitals of Birmingham NHS Foundation Trust Birmingham UK

**Keywords:** fragility fracture, obstructive sleep apnoea, type 2 diabetes

## Abstract

**Objective:**

This study examines the risk of fragility fractures in patients with type 2 diabetes (T2D) who have obstructive sleep apnoea (OSA) compared to those without OSA.

**Method:**

This retrospective cohort study uses UK primary care data from 2000 to 2022. T2D patients without prior fragility fractures were included. Patients with OSA were matched with up to 4 non‐OSA patients based on age, sex, BMI and T2D duration. Two cohorts were analysed based on whether OSA was diagnosed before (cohort 1) or after (cohort 2) the T2D diagnosis. Hazard ratios were calculated using a Cox proportional hazards model.

**Results:**

Cohort 1 included 19,795 patients with OSA and 64,124 without OSA. Cohort 2 included 21,769 patients with OSA and 66,350 without OSA. OSA was associated with an increased risk of incident fragility fractures in both cohorts [aHR (95% CI): 1.12 (1.00–1.25); *p* = 0.04, and 1.15 (1.05–1.26; *p* = 0.002), respectively]. The point estimates of the subgroup analysis by age and gender of cohort 1 suggest an association between OSA and the risk of fractures in men and in those above 50 years old. In cohort 2, the subgroup analysis point estimate suggests an association between OSA and fragility fractures in men and women and those above and below 50 years old.

**Conclusion:**

OSA is associated with an increased fragility fracture risk in T2D patients. Further studies are needed to determine if treating OSA reduces this risk. Clinicians should consider bone health and fracture risk, particularly for older patients.

## Graphical abstract

Association between obstructive sleep aponea (OSA) and risk of fragility fractures in patients with type 2 diabetes (T2D). This population‐based retrospective study demonstrates that the presence of OSA may be associated with an increased risk of fragility fractures among patients with T2D.

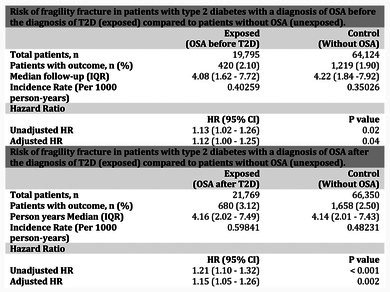

## 1 Introduction

The burden of fragility fractures is increasing rapidly due to the ageing population. These fractures are associated with substantial economic costs, long‐term disability and increased morbidity and mortality [1, 2]. Globally, it is estimated that 40%–50% of women and 13%–22% of men will experience fragility fractures in their lifetime [3]. In the United Kingdom, fragility fractures impose a considerable economic burden on the healthcare system and social services, with approximately 549,000 new fragility fracture cases reported annually [4]. The annual cost of managing fragility fractures for the National Health Service (NHS) is estimated at around £4.7 billion [4].

Despite having higher bone density and a high body mass index (BMI), individuals with type 2 diabetes (T2D) face an increased risk of fragility fractures. Individuals with T2D have a 1.2 to 1.7‐fold increased risk of hip and other fragility fractures compared to non‐diabetic individuals [5]. This increased risk is due to various factors, including poor glycaemic control, medication use (such as insulin, canagliflozin and glitazones), episodes of hypoglycaemia, oxidative stress, complications of T2D (such as chronic kidney disease), and the accumulation of advanced glycation end products, all of which lead to changes in bone metabolism and structure [6, 7, 8, 9]. Considering the significant burden of T2D and fragility fractures, it is crucial to identify modifiable risk factors to reduce the burden of T2D.

Obstructive sleep apnoea (OSA) is very common in patients with T2D, with a prevalence range from 6% to 86% [10]. OSA has been linked to adverse metabolic outcomes, including hypertension, glucose dysregulation, atherosclerosis and dyslipidemia, as well as cardiovascular disease, microvascular complications and oxidative and nitrosative stress [11, 12]. Recent evidence suggests potential links between intermittent hypoxia, sleep disorders and impaired bone health [13, 14]. In OSA, repetitive episodes of upper airway obstruction result in tissue ischaemia, oxidative stress, acidosis and decreased vascular perfusion. These physiological changes can negatively impact bone remodelling by disrupting osteoblast and osteoclast function, thereby compromising bone microarchitecture and reducing bone mineral density [15, 16]. Additionally, OSA is associated with disrupted sleep and lower serum vitamin D levels, further contributing to the deterioration of bone health [14]. These adverse effects are worsened by decreased physical activity and increased sedentary time, which directly impair bone health and significantly elevate the risk of fragility fractures. Given the high prevalence of T2D and OSA and their frequent co‐occurrence, the presence of shared pathophysiological mechanisms may exacerbate bone deterioration, potentially leading to increased incidence of fragility fractures. Evaluating the association between OSA and fragility fractures in patients with T2D is crucial for improving the risk of fragility fractures and guiding clinical management to reduce fracture‐related morbidity and healthcare costs.

Given the high prevalence of T2D and OSA, and their frequent co‐occurrence, the presence of shared pathophysiological mechanisms may exacerbate bone deterioration, potentially leading to an increased incidence of fragility fractures. Evaluating the association between OSA and fragility fractures in patients with T2D is crucial for improving the identification of fragility fractures and guiding clinical management to reduce fracture‐related morbidity and healthcare costs. Hence, we hypothesised that this study explicitly examined whether the timing of OSA diagnosis—either before or after the onset of T2D—had any impact on the risk of fragility fractures. To test this hypothesis, our study aims to assess OSA as a risk factor for fragility fractures in patients with T2D using a matched population‐based retrospective cohort study.

## 2 Research Design and Methods

### 2.1 Study Design

A population‐based, retrospective, matched open cohort study was conducted from January 1, 2000, to April 1, 2022, to assess the risk of fragility fractures in patients with OSA compared to randomly matched patients without OSA within a base cohort of patients with T2D.

### 2.2 Data Source

Data were extracted from the Clinical Practice Research Datalink (CPRD) Aurum, a real‐world primary care database containing anonymised medical records of 41 million patients, including 13 million currently registered in a network of over 1491 primary care practices across the U.K. [17]. The CPRD database includes coded and anonymised electronic health record (EHR) data from primary care practices on demographics, diagnoses and symptoms, drug prescriptions, vaccination history, laboratory tests and patient referrals [18]. It is widely utilised for epidemiological research and has been used in over 2000 observational studies in peer‐reviewed journals [19]. CPRD data have also been employed in numerous studies in the context of OSA, T2D or fragility fractures [20, 21, 22].

### 2.3 Ethics

Data collection and research utilising anonymised CPRD data received ethical approval from the National Research Ethics Services Committee (NRES). The study underwent independent scientific review to obtain approval for research using CPRD data. The Scientific Review Committee approved the use of CPRD data for this study in June 2022 (reference 22–001848).

### 2.4 Study Population

Adults aged ≥ 18 years with a diagnosis of T2D were eligible for inclusion in this study. The diagnosis of T2D was ascertained based on a diagnostic record using Snomed CT clinical codes (Code list S1 in the Data S1). To ensure adequate time to record all essential covariates and minimise the possibility of undiagnosed OSA before the diagnosis of T2D, eligibility was restricted to patients registered in general practice for at least 12 months. OSA diagnosis could be at any time prior to T2D diagnosis in cohort 1; diagnoses are captured in the CPRD data even if made before joining the practice. Patients with any pre‐existing fragility fracture were excluded from the study.

### 2.5 Exposed Cohort

The exposed cohort consisted of patients diagnosed with OSA, indicated by a record of OSA using a (Snomed CT) code alongside T2D (refer to Data S1, code list S2). There were two distinct groups of exposed patients of interest: (1) patients with a diagnosis of OSA before the incident diagnosis of T2D and (2) patients with a diagnosis of OSA after their T2D diagnosis.

### 2.6 Unexposed Cohort

For each exposed patient diagnosed with OSA before the incident diagnosis of T2D, up to four unexposed patients without OSA were directly matched for age (±1 year), sex, and BMI (±2 kg/m^2^) at the time of T2D diagnosis. Matched unexposed patients were randomly selected from a pool of eligible patients without OSA, without replacement. The index date was the date of T2D diagnosis for both the exposed and the unexposed patients.

**FIGURE 1:**
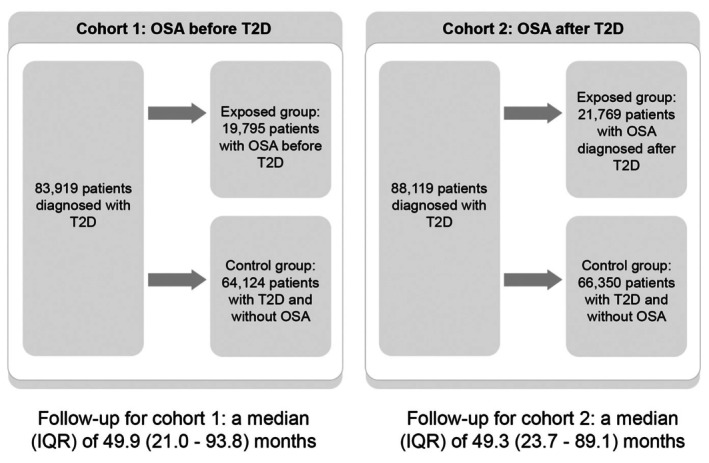
Flowchart of the study.

Similarly, for each exposed patient with an incident diagnosis of OSA after the diagnosis of T2D, up to four patients without OSA were directly matched by age (±1 year) at index date, sex, BMI (±2 kg/m^2^) and diabetes duration (±1 year). Matched unexposed patients were randomly selected from a pool of eligible patients without OSA, without replacement. The index date was defined as the date of OSA diagnosis for the exposed patients, and the same date was assigned to the matched unexposed patients to mitigate immortal time bias. A study flowchart is in Figure 1.

### 2.7 Follow‐Up Period

The follow‐up period for the included patients began on the index date (as defined in the previous section). The follow‐up end date (exit date) was determined by the earliest occurrence of one of the following events: the transfer date (when patients left the practice), the date the practice stopped contributing data to CPRD Aurum, the date of death, the date when the outcome (fragility fracture) was documented, or the study end date (1 April 2022).

### 2.8 Outcome and Covariates

The primary outcome of interest was fragility fractures, encompassing hip, wrist, spine and humerus fractures. The diagnosis of fragility fractures was determined using Snomed CT codes (code list S3 in the Data S1). Covariates were selected based on the literature [6] and biological plausibility, and were included in the regression analysis alongside the matching parameters. These covariates included age, sex, BMI, smoking status, ethnicity, alcohol consumption, Townsend deprivation quintile (a measure of social deprivation), hypoglycaemia, baseline HbA1c, Charlson Comorbidity Index (CCI), hyperthyroidism, renal function (eGFR), insulin use, glucose‐lowering agents, steroid use, bisphosphonates use, vitamin D and calcium supplement. Duration of diabetes was added to the covariates list for cohort 2. All diagnoses and measurements were the latest recorded prior to the index date, with no restriction; medications were those prescribed up to 60 days before the index date.

BMI was categorised as underweight/normal weight (< 25 kg/m^2^), overweight (25–30 kg/m^2^) and obese (≥ 30 kg/m^2^). Smoking status classified patients as non‐smokers, ex‐smokers and smokers. Social deprivation status was represented by the Townsend deprivation index, a scoring system with a maximum total score of 94, measuring 13 deprivation categories and divided into quintiles ranging from 1 to 5 (least to most deprived). Ethnicity was categorised as white, black, south Asian, mixed race and others. Missing values for BMI, smoking status and Townsend deprivation quintile were classified as separate missing categories within those variables.

### 2.9 Statistical Analysis

Standard descriptive statistics were used to summarise the baseline characteristics of the exposed and unexposed groups. Categorical variables were presented as frequencies and percentages, and continuous variables were displayed as means and standard deviations (SD) or medians and interquartile ranges (IQR), depending on the distribution of the variables. The Cox Proportional Hazards model was used to estimate the unadjusted and adjusted hazard ratios (HR) and 95% confidence intervals (CI) for the outcome fragility fractures, comparing the two sets of exposed and matched unexposed patient groups. The proportional hazards assumption was assessed using log–log plots and the Schoenfeld residual tests.

Sub‐group analyses were conducted, stratified by sex (male/female), age group (below and above 50 years of age) and combined age and gender groups (men < 50 years, men ≥ 50 years, women < 50 years and women ≥ 50 years).

All statistical analyses were performed using Stata/SE 17.0, and Dexter software was used for data extraction [23]. A *p*‐value < 0.05 was considered statistically significant.

## 3 Result

### 3.1 Cohort 1: Patients With OSA Diagnosis Before T2D and Their Matched Unexposed Controls

#### 3.1.1 Baseline Characteristics

A total of 83,919 patients with T2D were included in the study; 19,795 were in the exposed group (patients with OSA before T2D), and 64,124 patients were in the unexposed group (patients with T2D but without OSA). Baseline characteristics are summarised in Table 1. The study population was followed up for a median (IQR) of 49.9 (21.0–93.8) months. The overall study population was middle‐aged [mean (SD) = 57.2 (11.1) years] and predominantly male (74.8%). Most of the population had a BMI ≥ 30 kg/m^2^ (79.9%). The mean HbA1c was 7.4 (SD 1.9) %. The use of lipid‐lowering medications and antihypertensives was common across the entire study population but slightly higher in the exposed group. Patients in the exposed group also had higher rates of steroid and bisphosphonate use compared to those in the unexposed group (42.1% vs. 29.4% and 3.3% vs. 1.8%, respectively). Insulin use was similar in both groups (1.6% vs. 1.8%).

**TABLE 1:** Baseline characteristics of patients diagnosed with OSA before the diagnosis of T2D (exposed) and patients without OSA (unexposed).

	OSA (Exposed) (*n* = 19,795)	No OSA (Unexposed) (*n* = 64,124)
Age (years), mean (SD)	56.85 (11.5)	57.30 (10.9)
Age categories (years), *n* (%)		
18–29	219 (1.1)	323 (0.5)
30–39	1175 (5.9)	3280 (5.1)
40–49	3974 (20.1)	12,716 (19.9)
50*–*59	6752 (34.1)	22,413 (34.9)
60*–*69	5077 (25.7)	16,938 (26.4)
> 70	2598 (13.1)	8454 (13.2)
Sex categories, *n* (%)		
Male	14,751 (74.5)	48,037 (74.9)
Female	5044 (25.5)	16,087 (25.1)
BMI (kg/m^2^), mean (SD)	38.4 (8.2)	36.5 (6.8)
BMI categories, *n* (%)		
< 25 kg/m^2^	345 (1.7)	1392 (2.2)
25–30 kg/m^2^	2240 (11.3)	8891 (13.9)
> 30 kg/m^2^	16,450 (83.1)	50,373 (78.6)
Missing	760 (3.8)	3468 (5.4)
Townsend deprivation quintile, *n* (%)		
1 Least deprived	3168 (16.0)	10,017 (15.6)
2	3.581 (18.1)	11,361 (17.7)
3	3708 (18.8)	12,066 (18.8)
4	4223 (21.3)	14,032 (21.9)
5 Most deprived	4747 (23.9)	15,630 (24.4)
Missing	368 (1.9)	1018 (1.6)
Hypoglycemia	98 (0.5)	210 (0.3)
HbA1c %, mean (SD)	7.26 (1.8)	7.41 (1.9)
HbA1c categories, *n* (%)		
≤ 6.5%	2447 (12.4)	7068 (11.0)
6.5%–7.5%	1990 (10.1)	5927 (9.2)
7.5%–8.5%	567 (1.9)	1769 (2.8)
≥ 8.5%	955 (4.8)	3417 (5.3)
Smoker categories, *n* (%)		
Non‐smoker	4089 (20.7)	15,816 (24.7)
Ex‐smoker	9, 093 (45.9)	27,401 (42.7)
Current Smoker	6307 (31.9)	19,551 (30.5)
Missing	306 (1.6)	1356 (2.1)
Alcohol use, *n* (%)	1382 (6.9)	4074 (6.4)
Ethnicity, *n* (%)		
White	15,255 (77.1)	47,613 (74.3)
Black	784 (3.9)	3258 (5.1)
South Asian	1441 (7.3)	5050 (7.9)
Mixed race	527 (0.8)	153 (0.8)
Others	205 (1.0)	664 (1.0)
Missing	1957 (9.9)	7, 012 (10.9)
eGFR (mL/min/1.73 m^2^), mean (SD)	85.8 (19.3)	86.3 (18.4)
eGFR category, *n* (%)		
> 90 (stage 1)	8258 (41.7)	26,952 (42.0)
60–89 (stage 2)	8451 (42.7)	27,075 (42.2)
30–59 (stage 3)	1682 (8.5)	4625 (7.2)
< 30 (stage4)	103 (0.5)	224 (0.4)
Missing	1301 (6.6)	5248 (8.2)
Charlson Comorbidity Index, mean (SD)	2.34 (2.2)	2.63 (2.4)
Baseline comorbidities, *n* (%)		
Graves or hyperthyroidism	304 (1.5)	679 (1.1)
Chronic kidney disease	1294 (6.5)	3260 (5.1)
Stroke/TIA	1240 (6.3)	3045 (4.8)
Metastatic cancer	46 (0.2)	183 (0.3)
Baseline drug use (within 60 days prior to index date), *n* (%)		
Lipid‐lowering drugs	10, 172 (51.4)	29,452 (45.9)
Glucose‐lowering drugs	6639 (33.5)	21,046 (32.8)
Antihypertensive drugs	13.316 (67.3)	38,238 (59.6)
Systemic steroids	8337 (42.1)	18,876 (29.4)
Bisphosphonate	662 (3.3)	1151 (1.8)
Insulin	308 (1.6)	1178 (1.8)
Calcium supplements	1972 (9.9)	4033 (6.3)
Vitamin D	3774 (19.1)	8201 (12.8)

*Note:* Continuous data are presented as median (IQR) or mean (SD). Categorical variables are presented as *n* (%). Townsend deprivation index is a composite score with a maximum total score of 94 to measure 13 deprivation categories. This index is categorised into quintiles ranging from most least to most deprived, and it is widely used in CPRD studies.

Abbreviations: BMI, body mass index; eGFR, estimated glomerular filtration rate; HbA1c, haemoglobin A1C; OSA, obstructive sleep apnoea; TIA, transient ischaemic attack.

#### 3.1.2 The Risk of Fragility Fractures

During the observation period, a total of 420 (2.1%) fragility fractures were documented in patients with OSA and 1219 (1.9%) in patients without OSA. Patients with T2D and OSA diagnosis (exposed group) were significantly more likely to have an incident code for fragility fractures compared to patients without OSA, with a crude HR of 1.13 (95% CI 1.02–1.26; *p* = 0.02). After adjustment for age, sex, BMI, smoking status, alcohol, Townsend deprivation index, ethnicity, eGFR, hypoglycaemia, HbA1c, CCI, hyperthyroidism, glucose‐lowering agents, insulin use, steroid use, bisphosphonates use, vitamin D and calcium supplement, this finding remained similar, with an adjusted HR of 1.12 (95% CI 1.00–1.25; *p* = 0.04; Table 2). The full regression analysis results are in Table S1.

**TABLE 2:** Risk of fragility fracture in patients with type 2 diabetes with a diagnosis of OSA before the diagnosis of T2D (exposed) compared to patients without OSA (unexposed).

	Exposed (OSA before T2D)	Control (Without OSA)
Total patients, *n*	19,795	64,124
Patients with outcome, *n* (%)	420 (2.10)	1219 (1.90)
Median follow‐up (IQR)	4.08 (1.62–7.72)	4.22 (1.84–7.92)
Incidence rate (Per 1000 person‐years)	0.40259	0.35026
**Hazard ratio**		
	**HR (95% CI)**	***p***
Unadjusted HR	1.13 (1.02–1.26)	0.02
Adjusted HR	1.12 (1.00–1.25)	0.04
**Sub‐group analysis based on sex**		
Adjusted HR: Male	1.19 (1.03–1.36)	0.01
Adjusted HR: Female	1.01 (0.83–1.22)	0.89
**Sub‐group analysis based on age**		
Adjusted HR: Aged ≥ 50	1.12 (0.99–1.25)	0.06
Adjusted HR: Aged < 50	0.89 (0.66–1.21)	0.46
**Combined sex‐age analysis**		
Adjusted HR: Female < 50	0.84 (0.49–1.43)	0.52
Adjusted HR: Female ≥ 50	0.99 (0.81–1.22)	0.99
Adjusted HR: Male < 50	0.93 (0.65–1.34)	0.69
Adjusted HR: Male ≥ 50	1.19 (1.02–1.37)	0.02

*Note:* *p* values derived from Cox regression. Adjusted for age, sex, body mass index, smoking, Townsend, ethnicity, insulin use, glucose‐lowering agents, steroid use, bisphosphonates use, vitamin D, calcium supplement, alcohol, eGFR, Hypoglycaemia, HbA1c, Carlson Comorbidity Index and hyperthyroidism.

Abbreviations: CI, confidence interval; HR, hazard rate ratio; IQR, intra quantile range.

The age and sex‐stratified analyses are presented in Table 2. The associations between OSA and incident fractures were observed mainly in men (adjusted HR 1.19, 95% CI 1.03–1.36; *p* = 0.01) and people aged ≥ 50 years [adjusted HR 1.12 (CI % 0.99–1.25; *p* = 0.06)].

In the combined age‐sex‐stratified analysis (Table 2), only the males aged ≥ 50 years subgroup had a significantly increased risk of incident fragility fractures in those with OSA, adjusted HR 1.19 (95% CI 1.02–1.37; *p* = 0.02).

### 3.2 Cohort 2: Patients With OSA Diagnosis After T2D Diagnosis and Their Matched Unexposed Patients

#### 3.2.1 Baseline Characteristics

In this cohort, a total of 88,119 patients with T2D were included, with 21,769 patients in the exposed group (patients with OSA diagnosed after T2D) and 66,350 patients in the unexposed group (patients without OSA but with T2D). Baseline characteristics are summarised in Table 3. The study population was followed up for a median (IQR) of 49.3 (23.7–89.1) months. The results are similar to those observed in patients with OSA before T2D. However, unlike cohort 1, insulin use was higher in the exposed group (patients with OSA diagnosed after T2D) compared to the unexposed group (patients without OSA) (25.3% vs. 15.4%).

**TABLE 3:** Baseline characteristics of patients diagnosed with OSA after the diagnosis of T2D (exposed) and patients without OSA (unexposed).

	Exposed (*n* = 21,769)	Unexposed (*n* = 66,350)
Age (years), mean (SD)	59.65 (11.4)	60.27 (10.9)
Age categories (years), *n* (%)		
18–29	137 (0.6)	144 (0.2)
30*–*39	892 (4.1)	2121 (3.2)
40*–*49	3281 (15.1)	9454 (14.3)
50–59	6712 (30.8)	20,711 (31.2)
60*–*69	6576 (30.2)	20,790 (31.3)
%> 70	4171 (19.2)	13,130 (19.8)
Sex categories, *n* (%)		
Male	14,727 (67.7)	45,690 (68.9)
Female	7042 (32.4)	20,660 (31.1)
BMI (kg/m^2^), mean (SD)	38.4 (8.1)	36.4 (6.5)
BMI categories, *n* (%)		
< 25 kg/m^2^	435 (2.0)	1569 (2.4)
25–30 kg/m^2^	2430 (11.2)	9347 (14.1)
> 30 kg/m^2^	18,380 (84.4)	53,992 (81.4)
Missing	524 (2.4)	1442 (2.2)
Townsend deprivation quintile, *n* (%)		
1	3005 (13.8)	9593 (14.5)
2	3678 (16.9)	11,575 (17.5)
3	4029 (18.5)	12,651 (19.1)
4	4904 (22.5)	14,756 (22.2)
5	5745 (26.4)	16,776 (25.3)
Missing	408 (1.9)	999 (1.5)
Hypoglycemia	806 (3.7)	1593 (2.4)
Diabetes duration	7.61 (7.75)	6.90 (6.03)
HbA1c % mean (SD)	7.60 (1.6)	7.48 (1.6)
HbA1c categories, *n* (%)		
≤ 6.5%	3867 (17.76)	12,794 (19.28)
6.5%–7.5%	4564 (20.97)	14,015 (21.12)
7.5%–8.5%	3383 (20.95)	6861 (10.34)
≥ 8.5%	3073 (14.12)	8226 (12.40)
Missing	7882 (36.21)	24,454 (36.86)
Smoker categories, *n* (%)		
Non‐smoker	3987 (18.32)	14,540 (21.91)
Ex‐smoker	11,232 (51.60)	31,814 (47.95)
Smoker	6379 (29.30)	19,294 (29.08)
Missing	171 (0.79)	702 (1.06)
Alcohol use, *n* (%)	1479 (6.79%)	4137 (6.24)
Ethnicity, *n* (%)		
White	16,186 (74.35)	48,109 (72.51)
Black	1052 (4.83)	3792 (5.72)
South Asian	2020 (9.28)	5934 (8.94)
Mixed race	160 (0.73)	558 (0.84)
Others	225 (1.03)	678 (1.02)
Missing	2126 (9.77)	7279 (10.97)
T2D duration, mean (SD)	7.6 (7.6)	6.9 (6.04)
eGFR (mL/min/1.73 m^2^), mean (SD)	80.32 (23.2)	82.69 (20.8)
eGFR category, *n* (%)		
> 90 (stage1)	8123 (37.3)	25,880 (39.0)
60–89 (stage2)	8641 (39.7)	28,190 (42.5)
30–59 (stage 3)	3325 (15.3)	7851 (11.8)
< 30 (stage4)	590 (2.7)	967 (1.5)
Missing	1090 (5.0)	3462 (5.2)
Charlson Comorbidity Index, mean (SD)	4.38 (2.6)	4.08 (2.4)
Baseline comorbidities, *n* (%)		
Primary hyperparathyroidism	350 (1.6)	859 (1.3)
Chronic kidney disease	3138 (14.4)	7330 (11.1)
Stroke/TIA	1971 (9.1)	4260 (6.4)
Metastatic cancer	38 (0.2)	189 (0.3)
Baseline drug use (within 60 days of index), *n* (%)		
Lipid‐lowering drugs	17,965 (82.5)	51,992 (78.4)
Glucose‐lowering drugs	18,191 (83.6)	54,085 (81.5)
Antihypertensive drugs	18,432 (84.7)	51,367 (77.4)
Systemic steroids	9794 (44.9)	20,749 (31.3)
Bisphosphonate	860 (3.9)	1448 (2.2)
Insulin	5511 (25.3)	10,247 (15.4)
Calcium supplements	3047 (14.0)	5954 (8.9)
Vitamin D	5292 (24.3)	10,460 (15.8)

*Note:* Continuous data are presented as median (IQR) or mean (SD). Categorical variables are presented as *n* (%). Townsend deprivation index is a composite score with a maximum total score of 94 to measure 13 deprivation categories. This index is categorised into quintiles ranging from most least to most deprived, and it is widely used in CPRD studies.

Abbreviations: BMI, body mass index; eGFR, estimated glomerular filtration rate; HbA1c, haemoglobin A1C; OSA, obstructive sleep apnoea; TIA, transient ischaemic attack.

#### 3.2.2 The Risk of Fragility Fractures

In this cohort, a total of 680 (3.1%) fragility fractures were documented in patients with OSA and 1658 (2.5%) in patients without OSA. Patients with T2D with OSA diagnosis (exposed group) were significantly more likely to experience fragility fractures compared to patients without OSA, with an HR of 1.21 (95% CI 1.01–1.32; *p* < 0.001). After adjusting for the previously listed covariates, in addition to the duration of diabetes, the finding remained significant, with an adjusted HR of 1.15 (95% CI 1.05–1.26; *p* = 0.002; Table 4). The full regression analysis results are in Table S2.

**TABLE 4:** Risk of fragility fracture in patients with type 2 diabetes with a diagnosis of OSA after the diagnosis of T2D (exposed) compared to patients without OSA (unexposed).

	Exposed OSA after T2D	Control (without OSA)
Total patients, *n*	21,769	66,350
Patients with outcome, *n* (%)	680 (3.12)	1658 (2.50)
Person years, median (IQR)	4.16 (2.02–7.49)	4.14 (2.01–7.43)
Incidence rate (per 1000 person‐years)	0.59841	0.48231
**Hazard ratio**		
	**HR (95% CI)**	***p***
Unadjusted HR	1.21 (1.10–1.32)	< 0.001
Adjusted HR	1.15 (1.05–1.26)	0.002
**Sub‐group analysis based on sex**		
Adjusted HR: Male	1.12 (0.99–1.27)	0.05
Adjusted HR: Female	1.19 (1.05–1.37)	0.008
**Sub‐group analysis based on age**		
Adjusted HR: Aged ≥ 50	1.09 (0.99–1.20)	0.05
Adjusted HR: Aged < 50	1.14 (0.87 –1.51)	0.31
Combined Sex‐age analysis		
Adjusted HR: Female < 50	0.95 (0.61–1.48)	0.83
Adjusted HR: Female ≥ 50	1.17 (1.01–1.34)	0.03
Adjusted HR: Male < 50	1.33 (0.93 –1.89)	0.12
Adjusted HR: Male ≥ 50	1.04 (0.92–1.19)	0.47

*Note:* *p* values derived from Cox regression. Adjusted for age, sex, body mass index, smoking, Townsend, ethnicity, duration of diabetes, insulin use, glucose‐lowering agents, steroid use, bisphosphonates use, vitamin D, calcium supplement, alcohol, eGFR, Hypoglycaemia, HbA1c, Carlson Comorbidity Index and hyperthyroidism.

Abbreviations: CI, confidence interval; HR, hazard rate ratio; IQR, intra quantile range.

In the age and sex‐stratified analysis (Table 4), the association between OSA and incident fragility fractures was observed in men and women, and the point estimates suggest an association in those above and below 50 years of age.

In the combined age‐sex stratified analysis in Cohort 2, the effect estimates suggested an association between OSA and fragility fractures in females aged ≥ 50 years, males ≥ 50 years and males < 50 years, but the adjusted HR was only statistically significant in females ≥ 50 years [HRs 1.17 (95% CI 1.01–1.34; *p* = 0.03)].

## 4 Discussion

In this large population‐based study, we observed that patients with OSA and T2D were at increased risk of incident fragility fracture compared to patients with T2D but without OSA. This association was evident regardless of whether the OSA diagnosis preceded or followed the diagnosis of T2D. We also found that the observed associations might be modified by age and sex.

Research has increasingly focused on the potential impact of OSA on bone health, suggesting that OSA may be an independent risk factor for osteoporosis and fragility fractures in the general population, with limited attention given to people with T2D. Considering that OSA and T2D are co‐existing conditions primarily due to the shared risk factors and metabolic dysfunctions, it is plausible that these conditions may interact synergistically, potentially exacerbating impairments in bone health. A meta‐analysis of cohort studies reported a significant increase in the risk of osteoporosis among individuals with OSA, affecting both males and females with an OR of 1.92 (95% CI: 1.24–2.97) [24]. OSA was an independent factor contributing to lower bone mineral density (BMD) [15, 25]. One meta‐analysis concludes that individuals with OSA had significantly lower BMD in the lumbar spine (MD = −0.03; 95% CI −0.05, −0.01) and femur neck (MD = −0.06; 95% CI −0.12, 0.00). In addition, the risk of osteoporosis was higher in patients with OSA, with consistent findings across both men (OR = 2.03; 95% CI 1.23, 3.35) and women (OR = 2.56; 95% CI 1.96, 3.34) [26].

While there is limited data about the relationship between OSA and fragility fractures [27]. Our study contributes to this gap in knowledge by investigating the association between OSA and fragility fractures, specifically in T2D patients. Notably, our findings align with a recent retrospective study that examined the link between OSA severity and BMD in individuals with T2D. That study found low BMD and osteoporosis were prevalent in 59.4% of OSA patients. Furthermore, severe OSA was significantly associated with decreased BMD compared to those with mild OSA among T2D patients (*p* < 0.05), even after adjusting for confounding factors such as age, sex and BMI [28]. Another study showed that OSA is independently associated with an increased risk of vertebral fractures in women, with a hazard ratio (HR) of 2.00 [2]. This observed correlation between OSA severity, reduced BMD and osteoporosis supports our results. In contrast, a recent study examined the association between OSA and osteoporosis through bidirectional two‐sample Mendelian randomisation analysis. While the analysis revealed a significant causal association between OSA and reduced forearm‐BMD, implying that OSA affects bone metabolism, no causal association was found between OSA and fracture risk [29]. Nonetheless, the overall evidence highlights the complex interplay between OSA and bone health.

Several mechanisms might explain the observed links between OSA and incident fragility fractures in T2D. These mechanisms include intermittent hypoxia, changes in sleep patterns and disturbance in melatonin secretion. Additionally, OSA may lead to changes in hormonal regulation, such as activation of the hypothalamic–pituitary–adrenal (HPA) axis, suppression of growth hormone (GH) and insulin‐like growth factor 1 (IGF‐1) and dysregulation of sex hormones. Chronic inflammation, increased sympathetic nervous system activity, and changes in bone metabolism also play a role [30]. Furthermore, OSA could impact bone health indirectly by exacerbating comorbid conditions commonly associated with T2D, such as vitamin D deficiency, hypogonadism, obesity and insulin resistance and glucose intolerance, increasing the risk and severity of T2D [31].

Whether CPAP treatment can reduce the incidence of risk of fragility fractures in patients with T2D remains uncertain and warrants further investigation. However, CPAP has been shown to reduce the risk of falls, particularly in older people, and exerts favourable effects on several of the abovementioned mechanisms that underlie the association between OSA and incident fragility fractures [32, 33]. A recent study aimed to explore whether treating OSA patients with CPAP therapy could improve bone health. After 12 months of CPAP use, there was a significant increase in BMD, a rise in vitamin D and calcium levels, and a significant drop in parathyroid hormone (PTH) levels—indicating improved bone metabolism [34]. These mechanisms include improvements in hypoxia, sleep architecture, inflammation and regulating the HPA axis. Hence, it is plausible that CPAP could reduce the risk of fragility fractures in patients with T2D by addressing both direct and indirect factors that contribute to bone health.

In cohort 1 (patients with OSA diagnosed before T2D), the association between OSA and fragility fracture was primarily evident in men and those over 50 years of age. In cohort 2 (patients with OSA diagnosed after T2D), the association was evident in both males and females. Similar to cohort 1, the association was mainly in the group above 50 years of age. Differences in the association between OSA and incident fractures in the sex X age‐stratified analysis between cohorts 1 and 2 cannot be fully explained in this study. However, cohorts 1 and 2 are two different populations with different exposure times for OSA and T2D due to the study design (OSA before T2D and T2D after OSA). Such differences could contribute to the differences in the stratified analysis, especially since OSA is strongly linked to age and sex [35].

Our study has several strengths. We used a large cohort from a primary care database in the UK (CPRD Aurum), which has several advantages, including a large sample size, generalisability of the study's results to the UK population, and access to a wide range of covariates for adjustment [18]. Notably, this is the first study to examine whether OSA acts as a risk factor for fragility fracture in patients with T2D.

However, the study also has several limitations, primarily due to the retrospective and observational nature of the study design. Ascertainment of causality using real‐world data can be challenging, as potential confounding and the passive nature of outcome documentation may influence the findings. To address this, we attempted to minimise confounding by matching key variables and further adjusting for a comprehensive range of covariates in the analysis.

Another significant limitation is the potential for exposure misclassification bias, particularly due to the underdiagnosis of OSA within primary care settings. This underdiagnosis may attenuate the true effect sizes or potentially lead to a true association not being observed. In the UK, OSA diagnosis in primary care involves clinical assessments and patients' history, including investigation for comorbidities such as hypertension and T2D. Physical examination and the use of screening questionnaires, such as the ESS, are followed by referral to secondary care for a sleep study if OSA is suspected. Consequently, some patients in the unexposed group might have had undiagnosed OSA, with more severe and symptomatic cases being more likely to be diagnosed.

Moreover, the duration of OSA exposure was not accounted for in the current analysis, which may be an important factor influencing long‐term outcomes such as fracture risk. The diagnosis of OSA is established by a specialist and communicated to the patient's general practice for ongoing management and documentation. Although the potential for misdiagnosis exists, we expect such instances to be minimal. Additionally, data on OSA severity were not available, which limits our ability to assess dose–response relationships and represents an important area for future research.

Furthermore, we have not matched or adjusted for osteoporosis status; however, in the regression, we have adjusted for risk factors for fractures and osteoporosis.

Finally, it is also important to note that key lifestyle factors, such as physical inactivity and sedentary behaviour—both of which are known risk factors for T2D and fragility fractures—were not available in the CPRD dataset and thus could not be included in the analysis. The absence of these variables may result in residual confounding.

## 5 Conclusion

This study shows a significantly higher risk of fragility fractures in patients with T2D diagnosed with OSA compared to those without OSA, irrespective of whether OSA was diagnosed before or after T2D. Identifying and treating OSA may contribute to mitigating the elevated risk of fragility fractures in patients with T2D, but this needs to be examined in randomised controlled trials. This finding has potential implications for clinical practice, emphasising the importance of considering OSA as a modifiable risk factor for fragility fractures in individuals with T2D, focusing on high‐risk groups.

## Author Contributions

A.A.T., K.N. and N.J.A. conceived the study's idea. E.A.M. carried out the statistical analysis and wrote the first draft of the manuscript. A.S. and N.J.A. supported and reviewed the analysis. A.A.T., N.J.A., A.S. and K.N. supervised the study. All authors, E.A.M., A.S., K.N., N.J.A. and A.A.T., reviewed and revised the manuscript.

## Conflicts of Interest

A.A.T. is an employee and shareholder of Novo Nordisk. A.S. is an employee and shareholder of AstraZeneca. Novo Nordisk and AstraZeneca had no role in this project. The views expressed in the manuscript are those of the authors, not Novo Nordisk or AstraZeneca. All other authors report no conflicts of interest.

## Supporting information


**Data S1:** edm270100‐sup‐0001‐supinfo.docx.

## Data Availability

Data cannot be shared publicly because they were obtained under licence from CPRD. Data are available to researchers from CPRD subject to Research Data Governance approval.
