# Supplementary material for: Obstructive Sleep Apnoea and Risk of Fragility Fracture in Patients With Type 2 Diabetes: A Population‐Based Retrospective Cohort Study
Source: Endocrinol Diabetes Metab. 2025 Nov 7;8(6):e70100. doi: 10.1002/edm2.70100 (PMC12594633; doi:10.1002/edm2.70100)
Supplement: Supplementary file 1 — Data S1: edm270100‐sup‐0001‐supinfo.docx. [file EDM2-8-e70100-s001.docx]

## Supplementary file:

**Code list S1: Diagnosis of Type 2 Diabetes**

| **Snomed CT codes** | **Description** |
| --- | --- |
| 73466011 | Non-insulin dependent diabetes mellitus, |
| 197761014 | Type 2 diabetes mellitus, |
| 264679015 | Diabetic on diet only, |
| 264681018 | Diabetic on oral treatment, |
| 292576013 | Type II diabetes mellitus with multiple complications, |
| 292577016 | Type 2 diabetes mellitus with multiple complications, |
| 292579018 | Type 2 diabetes mellitus with ulcer, |
| 292581016 | Type II diabetes mellitus with ulcer, |
| 292582011 | Type II diabetes mellitus with gangrene, |
| 292583018 | Type 2 diabetes mellitus with gangrene, |
| 292589019 | Type II diabetes mellitus - poor control, |
| 292590011 | Type 2 diabetes mellitus - poor control, |
| 306113018 | Pre-existing diabetes mellitus, non-insulin-dependent, |
| 457328010 | Non-insulin-dependent diabetes mellitus without complication, |
| 457329019 | Type 2 diabetes mellitus without complication, |
| 457330012 | Type II diabetes mellitus without complication, |
| 459167016 | Type 2 diabetes mellitus with hypoglycaemic coma, |
| 459169018 | Type II diabetes mellitus with hypoglycaemic coma, |
| 459306016 | Type II diabetes mellitus with peripheral angiopathy, |
| 459308015 | Type 2 diabetes mellitus with peripheral angiopathy, |
| 459309011 | Non-insulin dependent diabetes mellitus with arthropathy, |
| 459310018 | Type 2 diabetes mellitus with arthropathy, |
| 459311019 | Type II diabetes mellitus with arthropathy, |
| 459312014 | Type II diabetes mellitus with neuropathic arthropathy, |
| 459313016 | Type 2 diabetes mellitus with neuropathic arthropathy, |
| 493773010 | NIDDM - Non-insulin dependent diabetes mellitus, |
| 493774016 | Type II diabetes mellitus, |
| 1223147012 | Insulin treated Type II diabetes mellitus, |
| 1223148019 | Insulin treated non-insulin dependent diabetes mellitus, |
| 1488898011 | Hyperosmolar non-ketotic state in type 2 diabetes mellitus, |
| 12336541000006114 | Retinopathy co-occurrent and due to type 2 diabetes mellitus, |
| 84601000006110 | Type 2 diabetes mellitus with polyneuropathy, |
| 280521000006117 | Non-insulin dependent diabetes mellitus with mononeuropathy, |
| 12702421000006111 | Type II diabetes mellitus with ophthalmic complications, |
| 12489851000006117 | Type II diabetes mellitus with exudative maculopathy, |
| 914031000006118 | Type 2 diabetes mellitus with renal complications, |
| 84521000006116 | Type 2 diabetes mellitus with hypoglycaemic coma, |
| 280591000006115 | Non-insulin-dependent diabetes mellitus with neuro comps, |
| 928541000006117 | Type 2 diabetes mellitus with persistent proteinuria, |
| 84591000006119 | Type 2 diabetes mellitus with peripheral angiopathy, |
| 85011000006119 | Type II diabetes mellitus without complication, |
| 928601000006118 | Type 2 diabetes mellitus with ketoacidotic coma, |
| 5110001000006118 | Pregnancy and type 2 diabetes mellitus, |
| 12702411000006115 | Type II diabetes mellitus with neurological complications, |
| 914161000006114 | Type II diabetes mellitus with retinopathy, |
| 84891000006116 | Type II diabetes mellitus with hypoglycaemic coma, |
| 7966941000006116 | Angina associated with type II diabetes mellitus, |
| 281211000006117 | Non-insulin dependent diabetes mellitus with gangrene, |
| 84911000006119 | Type II diabetes mellitus with multiple complications, |
| 914311000006114 | Type II diabetes mellitus with diabetic cataract, |
| 4758011000006112 | Type 2 diabetes mellitus with hyperosmolar coma, |
| 2288061000000111 | Type II diabetes mellitus in remission, |
| 914251000006116 | Type II diabetes mellitus with polyneuropathy, |
| 6959801000006112 | Diabetic cataract associated with type II diabetes mellitus, |
| 84981000006114 | Type II diabetes mellitus with renal complications, |
| 280531000006119 | Non-insulin dependent diabetes mellitus with nephropathy, |
| 84531000006118 | Type 2 diabetes mellitus with mononeuropathy, |
| 12336531000006116 | Retinopathy with type 2 diabetes mellitus, |
| 12702401000006118 | Mononeuropathy associated with type 2 diabetes mellitus, |
| 12363731000006113 | Polyneuropathy co-occurrent and due to type 2 diabetes mellitus, |
| 84471000006119 | Type 2 diabetes mellitus, |
| 84921000006110 | Type II diabetes mellitus with nephropathy, |
| 914151000006112 | Type 2 diabetes mellitus with retinopathy, |
| 6977971000006119 | Exudative maculopathy associated with type II diabetes mellitus, |
| 914081000006117 | Type II diabetes mellitus with neurological complications, |
| 1966311000006116 | Diabetic on oral treatment and glucagon-like peptide 1, |
| 914231000006111 | Type II diabetes mellitus with mononeuropathy, |
| 928581000006111 | Type 2 diabetes mellitus with ketoacidosis, |
| 84511000006112 | Type 2 diabetes mellitus with gangrene, |
| 6975221000006116 | Gangrene associated with type II diabetes mellitus, |
| 12702391000006115 | Diabetic cataract associated with type 2 diabetes mellitus, |
| 6985001000006110 | Peripheral circulatory disorder associated with type 2 diabetes mellitus, |
| 850691000006118 | Hyperosmolar non-ketotic state in type 2 diabetes mellitus, |
| 6969921000006119 | Neurologic disorder associated with type II diabetes mellitus, |
| 2460251000000115 | Diabetic on non-insulin injectable medication, |
| 12335671000006113 | Exudative maculopathy with type 2 diabetes mellitus, |
| 7966931000006114 | Angina associated with type 2 diabetes mellitus, |
| 84541000006111 | Type 2 diabetes mellitus with multiple complications, |
| 6951281000006117 | Kidney disorder associated with type 2 diabetes mellitus, |
| 12704971000006113 | Neurologic disorder associated with type 2 diabetes mellitus, |
| 12705081000006111 | Gastroparesis due to type 2 diabetes mellitus, |
| 840951000006119 | Insulin treated Type 2 diabetes mellitus, |
| 280581000006118 | Non-insulin-dependent diabetes mellitus with multiple comps, |
| 12336871000006115 | Disorder of eye with type 2 diabetes mellitus, |
| 6982511000006110 | Diabetic retinopathy associated with type II diabetes mellitus, |
| 12363681000006115 | Gastroparesis with type 2 diabetes mellitus, |
| 914391000006116 | Insulin treated Type 2 diabetes mellitus, |
| 84971000006111 | Type II diabetes mellitus with polyneuropathy, |
| 914261000006119 | Type 2 diabetes mellitus with nephropathy, |
| 84841000006113 | Type II diabetes mellitus, |
| 938331000006118 | Type II diabetes mellitus with exudative maculopathy, |
| 2288071000000116 | Type 2 diabetes mellitus in remission, |
| 914301000006111 | Type 2 diabetes mellitus with diabetic cataract, |
| 674961000006118 | NIDDM with peripheral circulatory disorder, |
| 84871000006117 | Type II diabetes mellitus with diabetic cataract, |
| 12334131000006118 | Neurological disorder co-occurrent and due to type 2 diabetes mellitus, |
| 1988741000006117 | Pre-existing type 2 diabetes mellitus in pregnancy, |
| 280551000006114 | Non-insulin dependent diabetes mellitus with ulcer, |
| 4796321000006113 | Pre-existing type 2 diabetes mellitus, |
| 12762301000006111 | Diabetic oculopathy associated with type 2 diabetes mellitus, |
| 6954121000006112 | Mononeuropathy associated with type II diabetes mellitus, |
| 84611000006113 | Type 2 diabetes mellitus with renal complications, |
| 928551000006115 | Type II diabetes mellitus with persistent proteinuria, |
| 85001000006117 | Type II diabetes mellitus with ulcer, |
| 6977421000006110 | Ketoacidosis in type II diabetes mellitus, |
| 587111000006111 | Non-insulin-dependent diabetes mellitus with ophthalm comps, |
| 84561000006110 | Type 2 diabetes mellitus with neurological complications, |
| 84941000006115 | Type II diabetes mellitus with neuropathic arthropathy, |
| 12704981000006111 | Diabetic retinopathy associated with type 2 diabetes mellitus, |
| 2359401000000111 | Conversion to non-insulin injectable medication, |
| 84641000006112 | Type 2 diabetes mellitus without complication, |
| 12336881000006117 | Disorder of eye co-occurrent and due to type 2 diabetes mellitus, |
| 281171000006119 | Non-insulin dependent diabetes mellitus - poor control, |
| 84491000006118 | Type 2 diabetes mellitus with arthropathy, |
| 938321000006116 | Type 2 diabetes mellitus with exudative maculopathy, |
| 84481000006116 | Type 2 diabetes mellitus - poor control, |
| 841351000006110 | Insulin treated Type II diabetes mellitus, |
| 12485441000006119 | NIDDM with peripheral circulatory disorder, |
| 1780981000006115 | Diabetic on non-insulin injectable, |
| 84861000006112 | Type II diabetes mellitus with arthropathy, |
| 914051000006113 | Type 2 diabetes mellitus with ophthalmic complications, |
| 12331051000006112 | Mononeuropathy with type 2 diabetes mellitus, |
| 84501000006114 | Type 2 diabetes mellitus with diabetic cataract, |
| 84571000006115 | Type 2 diabetes mellitus with neuropathic arthropathy, |
| 928561000006118 | Type 2 diabetes mellitus with persistent microalbuminuria, |
| 12331061000006114 | Mononeuropathy co-occurrent and due to type 2 diabetes mellitus, |
| 280561000006111 | Non-insulin-dependent d m with peripheral angiopath, |
| 12704991000006114 | Ketoacidotic coma in type 2 diabetes mellitus, |
| 84631000006119 | Type 2 diabetes mellitus with ulcer, |
| 84951000006118 | Type II diabetes mellitus with ophthalmic complications, |
| 914221000006113 | Type 2 diabetes mellitus with mononeuropathy, |
| 6983691000006114 | Diabetic oculopathy associated with type II diabetes mellitus, |
| 84851000006110 | Type II diabetes mellitus - poor control, |
| 928571000006113 | Type II diabetes mellitus with persistent microalbuminuria, |
| 7281511000006118 | Type 2 diabetes mellitus uncontrolled, |
| 281181000006116 | Non-insulin dependent d m with neuropathic arthropathy, |
| 6985011000006113 | Peripheral circulatory disorder associated with type II diabetes mellitus, |
| 1667891000000113 | Hyperosmolar non-ketotic state in type II diabetes mellitus, |
| 6981621000006113 | Persistent proteinuria associated with type II diabetes mellitus, |
| 914061000006110 | Type II diabetes mellitus with ophthalmic complications, |
| 84581000006117 | Type 2 diabetes mellitus with ophthalmic complications, |
| 84961000006116 | Type II diabetes mellitus with peripheral angiopathy, |
| 1667921000000117 | Type II diabetes mellitus with gastroparesis, |
| 1713231000006118 | QDiabetes (QDScore) type 2 diabetes 10-year risk, |
| 914041000006111 | Type II diabetes mellitus with renal complications, |
| 12704961000006118 | Diabetic oculopathy associated with type 2 diabetes mellitus, |
| 84621000006117 | Type 2 diabetes mellitus with retinopathy, |
| 280571000006116 | Non-insulin dependent diabetes mellitus, |
| 84881000006119 | Type II diabetes mellitus with gangrene, |
| 6975211000006112 | Gangrene associated with type 2 diabetes mellitus, |
| 281161000006114 | Non-insulin depend on diabetes mellitus with diabetic cataract, |
| 928591000006114 | Type II diabetes mellitus with ketoacidosis, |
| 12363691000006117 | Gastroparesis co-occurrent and due to type 2 diabetes mellitus, |
| 280541000006112 | Non-insulin dependent diabetes mellitus with polyneuropathy, |
| 4636411000006110 | Diet controlled diabetes mellitus, |
| 84991000006112 | Type II diabetes mellitus with retinopathy, |
| 914241000006118 | Type 2 diabetes mellitus with polyneuropathy, |
| 12335681000006111 | Exudative maculopathy co-occurrent and due to type 2 diabetes mellitus, |
| 299621000000117 | Type 2 diabetes mellitus with gastroparesis, |
| 6982151000006118 | Disorder due to type II diabetes mellitus, |
| 11931861000006112 | Pre-existing type 2 diabetes mellitus in pregnancy, |
| 12705011000006116 | Exudative maculopathy associated with type 2 diabetes mellitus, |
| 280511000006113 | Non-insulin dependent diabetes mellitus with hypoglyca coma, |
| 914071000006115 | Type 2 diabetes mellitus with neurological complications, |
| 84901000006117 | Type II diabetes mellitus with mononeuropathy, |
| 641581000006115 | Non-insulin-dependent diabetes mellitus with retinopathy, |
| 7966951000006119 | Diabetic angina pectoris associated with type 2 diabetes mellitus, |
| 6959091000006114 | Persistent microalbuminuria associated with type II diabetes mellitus, |
| 914271000006114 | Type II diabetes mellitus with nephropathy, |
| 84551000006113 | Type 2 diabetes mellitus with nephropathy, |
| 928611000006115 | Type II diabetes mellitus with ketoacidotic coma, |
| 587521000006111 | Non-insulin-dependent diabetes mellitus with renal comps, |
| 84931000006113 | Type II diabetes mellitus with neurological complications |

**Code list S2: Diagnosis of Obstructive Sleep Apnoea**

| **Snomed CT codes** | **Description** |
| --- | --- |
| 200354014 | Sleep apnoea, |
| 317034011 | [D]Insomnia with sleep apnoea, |
| 405691012 | [D]Hypersomnia with sleep apnoea, |
| 457106016 | [D]Sleep apnoea syndrome, |
| 1772611000006114 | Sleep hypoventilation, |
| 3693981000006111 | Sleep hypopnea, |
| 303181000006112 | [D]Syndrome sleep apnoea, |
| 267911000006119 | Obstructive sleep apnoea, |
| 3789311000006111 | Hypersomnia with sleep apnea, |
| 981761000006114 | Sleep apnea, |
| 11989401000006115 | Sleep apnoea syndrome, |
| 3694001000006117 | SAS - Sleep apnoea syndrome, |
| 12702791000006116 | Sleep apnoea syndrome, |
| 3773251000006110 | Obstructive sleep apnoea syndrome, |
| 7282631000006117 | Sleep related hypoventilation, |
| 3773281000006119 | OSA - Obstructive sleep apnea, |
| 3693961000006118 | Sleep apnea, |
| 3694011000006119 | Sleep hypopnoea, |
| 3693991000006114 | Sleep apnea syndrome, |
| 3693971000006113 | SAS - Sleep apnea syndrome, |
| 3773261000006112 | Obstructive sleep apnea syndrome, |
| 3773271000006117 | OSA - Obstructive sleep apnoea, |
| 3176551000006111 | Insomnia with sleep apnea, |
| 136581000006114 | Sleep apnoea, |
| 3773291000006116 | Obstructive sleep apnea, |
| 219501000000111 | Obstructive sleep apnoea |

**Code list S3: Fragility Fractures**

| **Snomed CT codes** | **Description** |
| --- | --- |
| **Hip Fracture** | |
| 10843018 | Fracture of neck of femur, |
| 107139013 | Fracture of acetabulum, |
| 277185016 | Closed reduction of fracture of hip, |
| 318655012 | Closed fracture acetabulum, anterior lip alone, |
| 318656013 | Closed fracture acetabulum, posterior lip alone, |
| 318657016 | Closed fracture acetabulum, anterior column, |
| 318658014 | Closed fracture acetabulum, posterior column, |
| 318659018 | Closed fracture acetabulum, floor, |
| 318660011 | Closed fracture acetabulum, double column transverse, |
| 318661010 | Closed fracture acetabulum, double column unspecified, |
| 318662015 | Other specified closed fracture acetabulum, |
| 318663013 | Closed fracture acetabulum NOS, |
| 318664019 | Open fracture acetabulum, anterior lip alone, |
| 318665018 | Open fracture acetabulum, posterior lip alone, |
| 318666017 | Open fracture acetabulum, anterior column, |
| 318667014 | Open fracture acetabulum, posterior column, |
| 318668016 | Open fracture acetabulum, floor, |
| 318669012 | Open fracture acetabulum, double column transverse, |
| 318670013 | Open fracture acetabulum, double column unspecified, |
| 318671012 | Other specified open fracture acetabulum, |
| 318672017 | Open fracture acetabulum NOS, |
| 319084014 | Closed fracture proximal femur, transcervical, |
| 319086011 | Closed fracture proximal femur, transepiphyseal, |
| 319087019 | Closed fracture proximal femur, midcervical section, |
| 319090013 | Closed fracture head of femur, |
| 319092017 | Closed fracture proximal femur, subcapital, Garden grade I, |
| 319093010 | Closed fracture proximal femur, subcapital, Garden grade II, |
| 319094016 | Closed fracture proximal femur, subcapital, Garden grade III, |
| 319095015 | Closed fracture proximal femur, subcapital, Garden grade IV, |
| 319100018 | Open fracture proximal femur, transcervical, |
| 319102014 | Open fracture proximal femur, transepiphyseal, |
| 319103016 | Open fracture proximal femur, midcervical section, |
| 319108013 | Open fracture proximal femur,subcapital, Garden grade unspec, |
| 319109017 | Open fracture proximal femur,subcapital, Garden grade I, |
| 319110010 | Open fracture proximal femur,subcapital, Garden grade II, |
| 319111014 | Open fracture proximal femur,subcapital, Garden grade III, |
| 319112019 | Open fracture proximal femur,subcapital, Garden grade IV, |
| 319118015 | Closed fracture of proximal femur, pertrochanteric, |
| 319123015 | Closed fracture proximal femur, intertrochanteric, two part, |
| 319124014 | Closed fracture proximal femur, subtrochanteric, |
| 319128012 | Open fracture of proximal femur, pertrochanteric, |
| 319133011 | Open fracture proximal femur, intertrochanteric, two part, |
| 319134017 | Open fracture proximal femur, subtrochanteric, |
| 319135016 | Open fracture proximal femur, intertrochanteric, comminuted, |
| 319138019 | Pertrochanteric fracture, |
| 319140012 | Closed fracture of unspecified proximal femur, |
| 319141011 | Open fracture of unspecified proximal femur, |
| 319145019 | Open fracture of neck of femur NOS, |
| 358600013 | DHS - Dynamic hip screw primary fixation of neck of femur, |
| 391384012 | Hip fracture, |
| 391392015 | Subtrochanteric fracture, |
| 391393013 | Hip fracture NOS, |
| 391394019 | Open fracture base of neck of femur, |
| 393789014 | Hip pin for fixation of epiphysis, |
| 402869013 | Closed fracture proximal femur, other transcervical, |
| 402871013 | Open fracture proximal femur, other transcervical, |
| 402874017 | Closed fracture of neck of femur NOS, |
| 411305015 | Closed fracture of femur, subcapital, |
| 411306019 | Open fracture of femur, subcapital, |
| 411307011 | Closed fracture of femur, greater trochanter, |
| 411308018 | Closed fracture of femur, lesser trochanter, |
| 411309014 | Open fracture of femur, lesser trochanter, |
| 411310016 | Open fracture of femur, greater trochanter, |
| 1228040014 | Closed fracture acetabulum, |
| 1234136017 | Open fracture acetabulum, |
| 1485120014 | H/O hip fracture, |
| 2536030018 | Open fracture proximal femur, basicervical, |
| 3525917017 | Hip fracture probability score using fracture risk assessment tool, |
| 557961000006116 | Cl red intracaps frac neck femur fix-Garden cannulated screw, |
| 167971000006114 | Revisn to int fxn(no red) prox fem #+screw/nail device alone, |
| 210041000006119 | Primary cls red+int fxn prox fem #+screw/nail+plate device, |
| 5572321000006113 | Closed fracture of greater trochanter of femur, |
| 2819451000006111 | Closed fracture of intracapsular section of femur, |
| 299771000000110 | Closed fracture of femur, upper epiphysis, |
| 208731000006113 | Prim open reduct # neck femur & op fix - Richards screw, |
| 570551000006114 | Cls # prox femur, subcapital, Garden grade unspec., |
| 4849521000006117 | Closed fracture dislocation of hip joint, |
| 4843961000006119 | Open subtrochanteric fracture of femur, |
| 210941000006118 | Primary int fxn(no red) prox fem #+scrw/nail+intramed device, |
| 570541000006112 | Cls # prox femur, intracapsular section, unspecified, |
| 761181000006111 | Fixation of epiphysis using Adams hip pin, |
| 208591000006116 | Prim op red # nck femur & op fix - Deyerle multiple hip pin, |
| 257561000006119 | Open fracture proximal femur, transcervical, NOS, |
| 207591000006115 | Prmy open red+int fxn prox fem #+screw/nail+intramed device, |
| 5462241000006110 | Fracture of proximal end of femur, |
| 166821000006118 | Revision to ext fxn(without reduction) proximal femoral #, |
| 208741000006115 | Prim open reduct # neck femur & op fix - Ross Brown nail, |
| 167981000006112 | Revisn to int fxn(no red) prox fem #+screw/nail+plate device, |
| 370861000000115 | Delivery of rehabilitation for hip fracture, |
| 6518831000006111 | History of hip fracture, |
| 2702681000006114 | Open transcervical fracture of femur, |
| 258471000006114 | Open fracture of femur, intertrochanteric, |
| 6206851000006110 | Closed fracture of hip, |
| 750551000006113 | Late effect of fracture neck of femur, |
| 895691000006117 | #Neck of femur, |
| 5572301000006115 | Open subcapital fracture of femur, |
| 11923811000006113 | Cls # prox femur, intracapsular section, unspecified, |
| 208601000006112 | Prim op red # nck femur & op fix- Charnley compression screw, |
| 570571000006116 | Cls # proximal femur, trochanteric section, unspecified, |
| 256101000006113 | Open fracture-dislocation, hip joint, |
| 208561000006112 | Prim cls rd+int fxn prox fem #+screw/nail+intramdulry device, |
| 265421000006113 | Open # of proximal femur, trochanteric section, unspecified, |
| 564391000006113 | Closed fracture proximal femur, basicervical, |
| 793901000006115 | Fracture-dislocation or subluxation hip, |
| 166991000006112 | Revision to open red+ext fxtn of proximal femoral #, |
| 167991000006110 | Revsn to opn red+int fxtn prox fem #+screw/nail device alone, |
| 299791000000114 | Open fracture of femur, upper epiphysis, |
| 3032981000006117 | Closed fracture of acetabulum, |
| 210921000006113 | Primary int fxn(no red) prox fem #+screw/nail device alone, |
| 210031000006112 | Primary cls red+int fxn prox fem #+screw/nail device alone, |
| 567461000006115 | Closed reduction of intracapsular # NOF internal fixat DHS, |
| 5572381000006112 | Open fracture of greater trochanter of femur, |
| 207601000006111 | Prmy open red+int fxn prox femoral #+screw/nail device alone, |
| 2525641000006118 | Closed fracture of cervicotrochanteric section of femur, |
| 166801000006111 | Revision to closed reduction+ext fxn proximal femoral #, |
| 258191000006119 | Open fracture of proximal femur, pertrochanteric, NOS, |
| 564491000006115 | Closed fracture proximal femur, transcervical, NOS, |
| 159511000006113 | Rvsn to opn red+int fxtn prox fem #+ scrw/nail+plate device, |
| 570621000006118 | Cls red+int fxn proximal femoral #+screw/nail device alone, |
| 208701000006117 | Prim open reduct # neck femur & op fix - Massie nail plate, |
| 565451000006113 | Closed fracture-dislocation, hip joint, |
| 6219701000006112 | Open fracture of hip, |
| 4849551000006114 | Closed fracture subluxation of hip joint, |
| 3762541000006110 | Open fracture of acetabulum, |
| 159501000006110 | Rvsn to int fxn(no red) prox fem #+screw/nail+intramed dev, |
| 5572281000006119 | Closed subcapital fracture of femur, |
| 7641181000006112 | Closed reduction and screw of fracture of proximal femur, |
| 5572361000006119 | Open fracture of lesser trochanter of femur, |
| 210531000006116 | Primary external fixation(without reduction) prox femoral #, |
| 210151000006118 | Primary cls reduction+external fixation proximal femoral #, |
| 5462341000006115 | Open fracture of base of neck of femur, |
| 208691000006117 | Prim open reduct # neck femur & op fix - Jewett nail plate, |
| 159491000006119 | Rvsn to cls red+int fxn prox fem #+screw/nail+plate device, |
| 2593851000006113 | Femoral neck fracture, |
| 562981000006117 | Closed fracture of femur, intertrochanteric, |
| 259461000006112 | Open fracture head, femur, |
| 4849541000006112 | Open fracture dislocation of hip joint, |
| 5133001000006116 | Closed reduction and internal fixation of proximal femoral fracture with Richard cannulated hip screw, |
| 255691000006116 | Open fracture-subluxation, hip joint, |
| 208611000006110 | Prim op red # nck femur & op fix- Zickel intramed nail plate, |
| 208711000006119 | Prim open reduct # neck femur & op fix - Neufield nail plate, |
| 207611000006114 | Prmy open red+int fxn prox femoral #+screw/nail+plate device, |
| 44341000006111 | Opn # proximal femur, intracapsular section, unspecified, |
| 632911000006110 | Dynamic hip screw primary fixation of neck of femur, |
| 2593861000006110 | Fracture of hip, |
| 208681000006115 | Prim open reduct # neck femur & op fix - Holt nail, |
| 208641000006114 | Prim open red # neck femur & op fix - McLaughlin nail plate, |
| 570531000006119 | Cls # of proximal femur, pertrochanteric section, NOS, |
| 159521000006117 | Rvsn to opn red+int fxtn prox fem #+ scrw/nl+intramed device, |
| 2593871000006115 | NOF - Fracture of neck of femur, |
| 210931000006111 | Primary int fxn(no red) prox fem #+screw/nail+plate device, |
| 208721000006110 | Prim open reduct # neck femur & op fix - Pugh nail plate, |
| 208751000006118 | Prim open reduct # neck femur & op fix - Thornton nail plate, |
| 4843911000006117 | Closed subtrochanteric fracture of femur, |
| 5462321000006110 | Subtrochanteric fracture of femur, |
| 565211000006111 | Closed fracture, base of neck of femur, |
| 570611000006114 | Cls red+int fxn prox femoral #+Richard's cannulat hip screw, |
| 208671000006118 | Prim open reduct # neck femur & op fix - Blount nail plate, |
| 565801000006112 | Closed fracture-subluxation, hip joint, |
| 1992361000006117 | VTE risk assessment - hip fracture, |
| 4849561000006111 | Open fracture subluxation of hip joint, |
| 4843731000006114 | Closed fracture of head of femur, |
| 159351000006118 | Rvsn cls red+int fxn prox fem #+screw/nail+intramed device, |
| 159481000006117 | Rvsn to cls red+int fxn prox fem #+screw/nail device alone, |
| 5572341000006118 | Closed fracture of lesser trochanter of femur, |
| 570561000006111 | Cls # proximal femur, intertrochanteric, comminuted |
| **Humerus Fractur** | |
| 574014 | Fracture of upper end of humerus, |
| 66948017 | Closed reduction of fracture of humerus, |
| 84797011 | Fracture of shaft of humerus, |
| 110116013 | Fracture of humerus, |
| 318750014 | Closed fracture of proximal humerus, unspecified part, |
| 318751013 | Closed fracture proximal humerus, neck, |
| 318752018 | Closed fracture of proximal humerus, anatomical neck, |
| 318753011 | Closed fracture proximal humerus, greater tuberosity, |
| 318755016 | Closed fracture of humerus, upper epiphysis, |
| 318756015 | Closed fracture proximal humerus, three parts, |
| 318757012 | Closed fracture proximal humerus, four parts, |
| 318758019 | Closed fracture of proximal humerus not otherwise specified, |
| 318762013 | Open fracture of proximal humerus, unspecified part, |
| 318763015 | Open fracture proximal humerus, neck, |
| 318764014 | Open fracture of proximal humerus, anatomical neck, |
| 318767019 | Open fracture proximal humerus, greater tuberosity, |
| 318768012 | Open fracture proximal humerus, head, |
| 318769016 | Open fracture of humerus, upper epiphysis, |
| 318770015 | Open fracture proximal humerus, three parts, |
| 318771016 | Open fracture proximal humerus, four parts, |
| 318772011 | Open fracture of proximal humerus not otherwise specified, |
| 318773018 | Closed fracture of humerus, shaft or unspecified part, |
| 318774012 | Closed fracture of humerus NOS, |
| 318775013 | Closed fracture of humerus, shaft or unspecified part NOS, |
| 318777017 | Open fracture of humerus, shaft or unspecified part, |
| 318778010 | Open fracture of humerus NOS, |
| 318779019 | Open fracture of humerus, shaft or unspecified part NOS, |
| 318784013 | Closed fracture distal humerus, lateral condyle, |
| 318785014 | Closed fracture distal humerus, medial condyle, |
| 318786010 | Closed fracture of distal humerus, condyle(s) unspecified, |
| 318787018 | Closed fracture of distal humerus, trochlea, |
| 318788011 | Closed fracture distal humerus, lateral epicondyle, |
| 318789015 | Closed fracture distal humerus, capitellum, |
| 318790012 | Closed fracture distal humerus, bicondylar (T-Y fracture), |
| 318791011 | Closed fracture of distal humerus, multiple, |
| 318792016 | Closed fracture of distal humerus, not otherwise specified, |
| 318799013 | Open fracture of distal humerus, condyle(s) unspecified, |
| 318800012 | Open fracture of distal humerus, trochlea, |
| 318802016 | Open fracture distal humerus, lateral epicondyle, |
| 318803014 | Open fracture distal humerus, capitellum, |
| 318804015 | Open fracture distal humerus, bicondylar (T-Y fracture), |
| 318805019 | Open fracture of distal humerus, multiple, |
| 318806018 | Open fracture of distal humerus, not otherwise specified, |
| 318810015 | Fracture of humerus NOS, |
| 319071017 | Multiple fractures of clavicle, scapula and humerus, |
| 391345019 | Fracture of lower end of humerus, |
| 402854012 | Closed fracture of the distal humerus, |
| 402856014 | Open fracture of the distal humerus, |
| 455446019 | Closed multiple fractures of clavicle, scapula and humerus, |
| 455447011 | Open multiple fractures of clavicle, scapula and humerus, |
| 1222634011 | Closed fracture distal humerus, medial epicondyle, |
| 1223146015 | Open fracture of humerus, shaft, |
| 1229897016 | Closed fracture of the proximal humerus, |
| 1230477016 | Open fracture distal humerus, medial epicondyle, |
| 1231853014 | Closed fracture distal humerus, supracondylar, |
| 1232322015 | Open fracture distal humerus, supracondylar, |
| 1235549012 | Closed fracture of humerus, shaft, |
| 12702721000006118 | Open fracture distal humerus, medial condyle, |
| 3951431000006114 | Open fracture of upper arm, |
| 5461911000006114 | Fracture of distal end of humerus, |
| 3513461000006116 | Open supracondylar fracture of humerus, |
| 3578351000006111 | Fracture of upper arm, |
| 892461000006118 | Path. Fracture - upper arm, |
| 4396041000006119 | Fracture of proximal end of humerus, |
| 257781000006117 | Open fracture of the proximal humerus, |
| 4841951000006115 | Closed fracture of trochlea of humerus, |
| 259881000006117 | Open fracture distal humerus, medial condyle, |
| 5722041000006115 | Pathological fracture of humerus, |
| 4842001000006111 | Closed multiple fractures of lower end of humerus, |
| 2878911000006117 | Open fracture of shaft of humerus, |
| 3912011000006113 | Open fracture of upper end of humerus, |
| 399091000006110 | [X]Multiple fractures of clavicle, scapula and humerus, |
| 895501000006111 | #Humerus - lower end, |
| 990971000006114 | #Humerus NOS, |
| 6009811000006111 | Open multiple fractures of clavicle and/or scapula and/or humerus, |
| 3912021000006117 | Open fracture of proximal end of humerus, |
| 895481000006118 | #Humerus - upper end, |
| 3966821000006110 | Closed fracture of shaft of humerus, |
| 3451541000006117 | Closed supracondylar fracture of humerus, |
| 5510011000006110 | Closed fracture of distal end of humerus, |
| 895491000006115 | #Humerus - shaft, |
| 990961000006119 | #Humerus - shaft, |
| 3197601000006115 | Closed fracture of upper arm, |
| 5510031000006116 | Open fracture of lower end of humerus, |
| 564541000006113 | Closed fracture proximal humerus, head, |
| 892551000006119 | Fracture malunion - upper arm, |
| 12724251000006118 | Fracture of humerus NOS, |
| 5510001000006112 | Closed fracture of lower end of humerus, |
| 6009791000006112 | Closed multiple fractures of clavicle and/or scapula and/or humerus, |
| 895511000006114 | #Humerus NOS, |
| 5510041000006114 | Open fracture of distal end of humerus, |
| 4841801000006113 | Closed fracture of anatomical neck of humerus, |
| 11904951000006110 | Open fracture of the proximal humerus, |
| 12704271000006115 | Closed fracture distal humerus, medial condyle, |
| 3187271000006113 | Closed fracture of proximal end of humerus, |
| 5722031000006113 | Pathological fracture - upper arm, |
| 895471000006116 | #Humerus, |
| 12730461000006113 | Closed fracture of humerus, shaft or unspecified part, |
| 259861000006110 | Open fracture distal humerus, lateral condyle |
| **Distal Forearm Fracture** | |
| 125993015 | Fracture of radius AND ulna, |
| 133435011 | Open Colles' fracture, |
| 151267013 | Open fracture of forearm, |
| 151450016 | Closed fracture of forearm, |
| 277199017 | Closed reduction of fracture of radius and or ulna, |
| 318829014 | Closed fracture of radius, shaft, unspecified, |
| 318830016 | Closed fracture radius and ulna, middle, |
| 318831017 | Closed fracture of radius and ulna, shaft, NOS, |
| 318832012 | Open fracture of radius, shaft, unspecified, |
| 318833019 | Open fracture radius and ulna, middle, |
| 318834013 | Open fracture of radius and ulna, shaft, NOS, |
| 318838011 | Closed fracture of forearm, lower end, unspecified, |
| 318845011 | Closed fracture of ulna, lower epiphysis, |
| 318846012 | Closed fracture distal ulna, unspecified, |
| 318848013 | Smith's fracture - closed, |
| 318849017 | Closed Smith's fracture, |
| 318850017 | Closed Galeazzi fracture, |
| 318851018 | Closed volar Barton's fracture, |
| 318857019 | Closed fracture radial styloid, |
| 318858012 | Closed fracture distal radius, intra-articular, die-punch, |
| 318859016 | Closed fracture distal radius, extra-articular, other type, |
| 318862018 | Closed fracture distal radius, intra-articular, other type, |
| 318864017 | Closed fracture of forearm, lower end, NOS, |
| 318868019 | Open fracture of forearm, lower end, unspecified, |
| 318875018 | Open fracture of ulna, styloid process, |
| 318876017 | Open fracture of ulna, lower epiphysis, |
| 318877014 | Open fracture distal ulna - other, |
| 318880010 | Smith's fracture - open, |
| 318881014 | Open Galeazzi fracture, |
| 318882019 | Open volar Barton's fracture, |
| 318887013 | Open dorsal Barton's fracture, |
| 318888015 | Open fracture radial styloid, |
| 318889011 | Open fracture distal radius, intra-articular, die-punch, |
| 318890019 | Open fracture distal radius, extra-articular other type, |
| 318891015 | Open fracture distal radius, intra-articular other type, |
| 318893017 | Open fracture of forearm, lower end, NOS, |
| 318901010 | Closed fracture of radius and ulna, unspecified part, |
| 318902015 | Closed fracture of forearm, unspecified, |
| 318909012 | Closed fracture of radius and ulna, NOS, |
| 318910019 | Open fracture of radius and ulna, unspecified part, |
| 318911015 | Open fracture of forearm, unspecified, |
| 318912010 | Open fracture of radius (alone), unspecified, |
| 318913017 | Open fracture of ulna (alone), unspecified, |
| 318914011 | Open fracture of radius and ulna, NOS, |
| 318915012 | Fracture of radius and ulna, NOS, |
| 318936014 | Fracture at wrist and hand level, |
| 319074013 | Multiple fractures of forearm, |
| 319961016 | Closed fracture dislocation of wrist, |
| 319970018 | Open fracture dislocation wrist, |
| 322029018 | Sequelae of fracture at wrist and hand level, |
| 325399012 | [X]Fracture of other parts of forearm, |
| 325400017 | [X]Fracture of forearm, unspecified, |
| 391347010 | Fracture of radius NOS, |
| 391352017 | Fracture of shaft of radius, |
| 391354016 | Fracture of lower end of radius, |
| 391357011 | Fracture of ulna NOS, |
| 391360016 | Fracture of shaft of ulna, |
| 391366010 | Fracture of lower end of both ulna and radius, |
| 402214016 | Fracture of radius or ulna due to birth trauma, |
| 402859019 | Closed Colles' fracture, |
| 402860012 | Closed fracture of the distal radius, unspecified, |
| 402862016 | Open fracture of the distal radius, unspecified, |
| 402863014 | Closed fracture of radius (alone), unspecified, |
| 402864015 | Closed fracture of ulna (alone), unspecified, |
| 411337019 | Birth fracture of radius, |
| 411338012 | Birth fracture of ulna, |
| 451060018 | Open Barton's fracture, |
| 451061019 | Closed Barton's fracture, |
| 483745010 | Closed fracture of radius and ulna, shaft, |
| 485443016 | Closed fracture of radius and ulna, lower end, |
| 492363016 | Open fracture of radius and ulna, shaft, |
| 496729019 | Closed fracture of the radius and ulna, |
| 500272010 | Forearm fracture, |
| 507904017 | Open fracture of radius and ulna, lower end, |
| 1219674017 | Open fracture radial neck, |
| 1227930011 | Closed fracture of the radial shaft, |
| 1229710011 | Closed fracture of ulna, styloid process, |
| 1229927010 | Open fracture of the ulnar shaft, |
| 1231252012 | Closed fracture of the ulnar shaft, |
| 1232779012 | Open fracture of the radial shaft, |
| 1233086019 | Closed fracture of ulna, coronoid, |
| 1233093015 | Closed fracture radius, head, |
| 1233163011 | Open fracture radial head, |
| 1233512015 | Closed fracture radius, neck, |
| 1235130019 | Open fracture of ulna, coronoid, |
| 1490703015 | Closed fracture radius and ulna, distal, |
| 1495502013 | Wrist fracture - open, |
| 1495503015 | Open fracture radius and ulna, distal, |
| 1730651000000118 | Greenstick fracture of distal radius, |
| 3192421000006112 | Open fracture of radius, |
| 261551000006117 | Open dorsal Barton's fracture-subluxation, |
| 4842291000006112 | Closed dorsal Barton fracture, |
| 793501000006112 | Fracture of shafts of both ulna and radius, |
| 895521000006118 | #Radius/ulna, |
| 12726221000006118 | Fracture of radius and ulna, NOS, |
| 5971961000006118 | Open Barton fracture, |
| 256341000006113 | Open fracture-subluxation of the wrist, |
| 5507451000006115 | Fracture of radius and/or ulna due to birth trauma, |
| 11923781000006111 | Closed fracture of radius and ulna, shaft, NOS, |
| 12484111000006112 | Closed reduction of fracture of radius and or ulna, |
| 4842451000006117 | Open dorsal Barton fracture, |
| 256051000006113 | Open fracture-dislocation superior radio-ulnar joint, |
| 3629081000006117 | Open fracture of head of radius, |
| 4842431000006112 | Open dorsal Barton's fracture dislocation, |
| 3577351000006112 | Open fracture of shaft of radius, |
| 565411000006112 | Closed fracture-dislocation superior radio-ulnar joint, |
| 3678911000006117 | Closed fracture of neck of radius, |
| 569501000006112 | Closed volar Barton fracture-subluxation, |
| 3160991000006117 | Closed fracture of styloid process of ulna, |
| 4849001000006119 | Open fracture dislocation of distal radioulnar joint, |
| 565251000006112 | Closed fracture-dislocation distal radio-ulnar joint, |
| 3372201000006117 | Closed fracture of shaft of ulna, |
| 559791000006116 | Closd dorsal Barton's fracture, |
| 4848801000006115 | Closed fracture dislocation superior radioulnar joint, |
| 895581000006119 | #Radius/ulna NOS, |
| 3904161000006115 | Open fracture of coronoid process of ulna, |
| 2950731000006116 | Closed fracture of shaft of bone of forearm, |
| 4842271000006111 | Closed dorsal Barton's fracture subluxation, |
| 5462101000006115 | Fracture of shaft of radius and/or ulna, |
| 4842231000006113 | Closed volar Barton's fracture subluxation, |
| 387591000006110 | [X]Fracture of other & unspecified parts of wrist and hand, |
| 632631000006110 | Dupuytren's fracture, radius - closed, |
| 3784451000006118 | Open fracture of distal end of forearm, |
| 559861000006119 | Closed # radius neck, |
| 4842241000006115 | Closed volar Barton's fracture dislocation, |
| 4848921000006116 | Closed fracture dislocation radiocarpal joint, |
| 5971981000006111 | Closed Barton fracture, |
| 49851000006119 | Open volar Barton fracture-dislocation, |
| 4848881000006112 | Open fracture subluxation superior radioulnar joint, |
| 3034241000006111 | Closed fracture of lower end of radius AND ulna, |
| 991021000006118 | #Radius/ulna NOS, |
| 565741000006119 | Closed fracture-subluxation superior radio-ulnar joint, |
| 3143491000006113 | Open fracture of shaft of radius and ulna, |
| 565711000006118 | Closed fracture-subluxation radiocarpal joint, |
| 255671000006117 | Open fracture-subluxation, distal radio-ulnar joint, |
| 4842441000006119 | Open dorsal Barton's fracture subluxation, |
| 3189391000006111 | Open fracture of shaft of ulna, |
| 49861000006117 | Open volar Barton fracture-subluxation, |
| 895561000006112 | Colles fracture, |
| 5462121000006113 | Fracture of distal end of radius and ulna, |
| 4849021000006112 | Open fracture dislocation radiocarpal joint, |
| 4849171000006114 | Open fracture subluxation of distal radioulnar joint, |
| 257831000006114 | Open fracture of the radius and ulna, |
| 4842411000006118 | Open volar Barton fracture, |
| 54541000006116 | Wrist fracture - closed, |
| 4849091000006114 | Closed fracture subluxation of distal radioulnar joint, |
| 4849101000006115 | Closed fracture subluxation radiocarpal joint, |
| 4842401000006116 | Open volar Barton's fracture dislocation, |
| 4848861000006119 | Closed fracture subluxation superior radioulnar joint, |
| 256001000006114 | Open fracture-dislocation radiocarpal joint, |
| 569521000006119 | Closed volar Barton's fracture-dislocation, |
| 2950741000006114 | Closed fracture of shaft of radius and/or ulna, |
| 4198181000006116 | Open fracture of neck of radius, |
| 895551000006110 | #Radius/ulna-lower end-colles, |
| 851421000006114 | Torus fracture of radius, |
| 3070931000006119 | Closed fracture of distal end of forearm, |
| 4842391000006118 | Open volar Barton's fracture subluxation, |
| 565671000006117 | Closed fracture-subluxation of the wrist, |
| 561311000006117 | Closed dorsal Barton fracture-subluxation, |
| 256081000006117 | Open fracture-dislocation, distal radio-ulnar joint, |
| 3316131000006111 | Closed fracture of lower end of ulna, |
| 565381000006114 | Closed fracture-dislocation radiocarpal joint, |
| 2773201000006116 | Closed fracture of lower end of radius, |
| 5722051000006118 | Pathological fracture - forearm, |
| 2550381000006113 | Closed fracture of shaft of radius, |
| 895541000006113 | #Radius/ulna -shaft, |
| 12726211000006114 | Closed fracture distal ulna, unspecified, |
| 3932001000006116 | Open fracture of lower end of radius AND ulna, |
| 3619281000006119 | Closed fracture of coronoid process of ulna, |
| 561321000006113 | Closed dorsal Barton's fracture-dislocation, |
| 4848831000006111 | Open fracture dislocation superior radioulnar joint, |
| 255631000006115 | Open fracture-subluxation superior radio-ulnar joint, |
| 255581000006118 | Open fracture-subluxation radiocarpal joint, |
| 3619801000006110 | Closed fracture of head of radius, |
| 793921000006113 | Fracture-dislocation or subluxation of wrist, |
| 4849181000006112 | Open fracture subluxation radiocarpal joint, |
| 565781000006113 | Closed fracture-subluxation, distal radio-ulnar jt, |
| 12223181000006110 | Fracture of ulna due to birth trauma, |
| 261541000006119 | Open dorsal Barton's fracture-dislocation, |
| 4842281000006114 | Closed dorsal Barton's fracture dislocation, |
| 895571000006117 | Fracture of distal end of ulna, |
| 52291000006115 | Open Smith's fracture, |
| 5461991000006116 | Fracture of distal end of radius, |
| 632641000006117 | Dupuytren's fracture, radius - open, |
| 4842251000006118 | Closed volar Barton fracture, |
| 4688071000006117 | Closed reduction of fracture of radius and/or ulna, |
| 3572751000006110 | Forearm fracture |
| **Spinal Fractures.** | |
| 194542018 | Fracture of sacrum, |
| 194543011 | Fracture of coccyx, |
| 276467013 | Decompression of fracture of spine, |
| 276468015 | Complex decompression of fracture of spine, |
| 276469011 | Anterior decompression of fracture of spine, |
| 276470012 | Posterior decompression of fracture of spine, |
| 276471011 | Other specified decompression of fracture of spine, |
| 276474015 | Decompression of fracture of spine NOS, |
| 276481010 | Open reduction of fracture of spine NEC, |
| 276483013 | Spinal extension traction for fracture of spine, |
| 276484019 | Halo skull traction for fracture of spine, |
| 276485018 | Spinal traction for fracture of spine NEC, |
| 276487014 | Primary bedrest stabilisation of spinal fracture, |
| 276488016 | Primary collar stabilisation of spinal fracture, |
| 276491016 | Primary skull traction stabilisation of spinal fracture, |
| 276492011 | Primary cast stabilisation of spinal fracture, |
| 276495013 | Primary external fixation stabilisation of spinal fracture, |
| 276496014 | Primary other external stabilisation of spinal fracture, |
| 276498010 | Revision to bedrest stabilisation of spinal fracture, |
| 276501010 | Revision to collar stabilisation of spinal fracture, |
| 276502015 | Revision to skull traction stabilisation of spinal fracture, |
| 276505018 | Revision to cast stabilisation of spinal fracture, |
| 276508016 | Revision to other external stabilisation of spinal fracture, |
| 276510019 | Primary closed reduction spinal fracture alone, |
| 276522016 | Revision to closed reduction spinal fracture alone, |
| 276534012 | Primary open reduction spinal fracture alone, |
| 276543015 | Primary open reduction spinal fracture and external fixation, |
| 276546011 | Revision to open reduction spinal fracture alone, |
| 276559015 | Other specified other reduction of fracture of spine, |
| 276560013 | Other reduction of fracture of spine NOS, |
| 276566019 | Fixation of fracture of spine using Harrington rod, |
| 276582018 | Other specified fixation of fracture of spine, |
| 276583011 | Fixation of fracture of spine NOS, |
| 311305016 | Fatigue fracture of vertebra, |
| 318353013 | Closed fracture of unspecified cervical vertebra, |
| 318375010 | Closed fracture atlas, isolated arch or articular process, |
| 318376011 | Closed fracture atlas, comminuted, |
| 318377019 | Closed fracture axis, odontoid process, |
| 318378012 | Closed fracture axis, spondylolysis, |
| 318379016 | Closed fracture axis, spinous process, |
| 318380018 | Closed fracture axis, transverse process, |
| 318381019 | Closed fracture axis, posterior arch, |
| 318382014 | Closed fracture axis, tricolumnar, |
| 318383016 | Closed fracture cervical vertebra, burst, |
| 318384010 | Closed fracture cervical vertebra, wedge, |
| 318385011 | Closed fracture cervical vertebra, spondylolysis, |
| 318386012 | Closed fracture cervical vertebra, spinous process, |
| 318387015 | Closed fracture cervical vertebra, transverse process, |
| 318388013 | Closed fracture cervical vertebra, posterior arch, |
| 318389017 | Closed fracture cervical vertebra, tricolumnar, |
| 318390014 | Multiple closed fractures of cervical vertebrae, |
| 318391013 | Closed fracture of cervical spine not otherwise specified, |
| 318396015 | Open fracture of unspecified cervical vertebra, |
| 318422010 | Open fracture axis, spinous process, |
| 318426013 | Open fracture cervical vertebra, burst, |
| 318427016 | Open fracture cervical vertebra, wedge, |
| 318428014 | Open fracture cervical vertebra, spondylolysis, |
| 318429018 | Open fracture cervical vertebra, spinous process, |
| 318430011 | Open fracture cervical vertebra, transverse process, |
| 318431010 | Open fracture cervical vertebra, posterior arch, |
| 318432015 | Open fracture cervical vertebra, tricolumnar, |
| 318433013 | Multiple open fractures of cervical vertebrae, |
| 318434019 | Open fracture of cervical spine not otherwise specified, |
| 318435018 | Closed fracture thoracic vertebra, |
| 318436017 | Closed fracture thoracic vertebra, burst, |
| 318437014 | Closed fracture thoracic vertebra, wedge, |
| 318438016 | Closed fracture thoracic vertebra, spondylolysis, |
| 318439012 | Closed fracture thoracic vertebra, spinous process, |
| 318440014 | Closed fracture thoracic vertebra, transverse process, |
| 318441013 | Closed fracture thoracic vertebra, posterior arch, |
| 318442018 | Closed fracture thoracic vertebra, tricolumnar, |
| 318443011 | Other specified closed fracture thoracic vertebra, |
| 318445016 | Closed fracture thoracic vertebra not otherwise specified, |
| 318446015 | Open fracture thoracic vertebra, |
| 318447012 | Open fracture thoracic vertebra, burst, |
| 318448019 | Open fracture thoracic vertebra, wedge, |
| 318449010 | Open fracture thoracic vertebra, spondylolysis, |
| 318450010 | Open fracture thoracic vertebra, spinous process, |
| 318451014 | Open fracture thoracic vertebra, transverse process, |
| 318452019 | Open fracture thoracic vertebra, posterior arch, |
| 318453012 | Open fracture thoracic vertebra, tricolumnar, |
| 318454018 | Closed fracture lumbar vertebra, |
| 318455017 | Closed fracture lumbar vertebra, burst, |
| 318456016 | Closed fracture lumbar vertebra, wedge, |
| 318457013 | Closed fracture lumbar vertebra, spondylolysis, |
| 318458015 | Closed fracture lumbar vertebra, spinous process, |
| 318459011 | Closed fracture lumbar vertebra, transverse process, |
| 318460018 | Closed fracture lumbar vertebra, posterior arch, |
| 318461019 | Closed fracture lumbar vertebra, tricolumnar, |
| 318462014 | Open fracture lumbar vertebra, |
| 318463016 | Open fracture lumbar vertebra, burst, |
| 318464010 | Open fracture lumbar vertebra, wedge, |
| 318465011 | Open fracture lumbar vertebra, spondylolysis, |
| 318466012 | Open fracture lumbar vertebra, spinous process, |
| 318467015 | Open fracture lumbar vertebra, transverse process, |
| 318468013 | Open fracture lumbar vertebra, posterior arch, |
| 318469017 | Open fracture lumbar vertebra, tricolumnar, |
| 318473019 | Closed fracture sacrum, |
| 318474013 | Closed compression fracture sacrum, |
| 318475014 | Closed vertical fracture of sacrum, |
| 318476010 | Open fracture sacrum, |
| 318477018 | Open compression fracture sacrum, |
| 318478011 | Open vertical fracture of sacrum, |
| 318479015 | Closed fracture pelvis, coccyx, |
| 318480017 | Open fracture pelvis, coccyx, |
| 318482013 | Fracture of first cervical vertebra, |
| 318483015 | Fracture of second cervical vertebra, |
| 318484014 | Multiple fractures of cervical spine, |
| 318485010 | Fracture of lumbar spine and pelvis, |
| 318492017 | Multiple fractures of lumbar spine and pelvis, |
| 318493010 | Closed fracture of spine, unspecified,, |
| 318494016 | Open fracture of spine, unspecified,, |
| 318495015 | Fracture of spine without mention of spinal cord lesion NOS, |
| 318500017 | Closed fracture of cervical spine with cord lesion, |
| 318514017 | Open fracture of cervical spine with spinal cord lesion, |
| 318518019 | Open spinal fracture with central cervical cord lesion, C1-4, |
| 318524013 | Open spinal fracture with central cervical cord lesion, C5-7, |
| 318527018 | Open fracture of cervical spine with spinal cord lesion NOS, |
| 318528011 | Closed fracture of thoracic spine with spinal cord lesion, |
| 318542015 | Open fracture of thoracic spine with spinal cord lesion, |
| 318546017 | Open spinal fracture with central thoracic cord lesion, T1-6, |
| 318555019 | Open fracture of thoracic spine with spinal cord lesion NOS, |
| 318556018 | Closed fracture of lumbar spine with spinal cord lesion, |
| 318557010 | Closed spinal fracture with unspecified lumbar cord lesion, |
| 318558017 | Closed spinal fracture with complete lumbar cord lesion, |
| 318559013 | Closed spinal fracture with anterior lumbar cord lesion, |
| 318560015 | Closed spinal fracture with central lumbar cord lesion, |
| 318561016 | Closed spinal fracture with posterior lumbar cord lesion, |
| 318562011 | Closed spinal fracture with cauda equina lesion, |
| 318563018 | Open fracture of lumbar spine with spinal cord lesion, |
| 318564012 | Open spinal fracture with unspecified lumbar cord lesion, |
| 318565013 | Open spinal fracture with complete lumbar cord lesion, |
| 318566014 | Open spinal fracture with anterior lumbar cord lesion, |
| 318567017 | Open spinal fracture with central lumbar cord lesion, |
| 318568010 | Open spinal fracture with posterior lumbar cord lesion, |
| 318569019 | Open spinal fracture with cauda equina lesion, |
| 318571019 | Closed fracture of sacrum with spinal cord lesion, |
| 318573016 | Closed fracture of sacrum with complete cauda equina lesion, |
| 318574010 | Closed fracture of sacrum with other cauda equina injury, |
| 318575011 | Closed fracture of sacrum with other spinal cord injury, |
| 318580019 | Closed fracture of sacrum with spinal cord lesion NOS, |
| 318581015 | Open fracture of sacrum with spinal cord lesion, |
| 318582010 | Open fracture of sacrum with unspecified spinal cord lesion, |
| 318583017 | Open fracture of sacrum with complete cauda equina lesion, |
| 318584011 | Open fracture of sacrum with other cauda equina injury, |
| 318585012 | Open fracture of sacrum with other spinal cord injury, |
| 318586013 | Open fracture of sacrum with spinal cord lesion NOS, |
| 318587016 | Closed fracture of coccyx with spinal cord lesion, |
| 318589018 | Closed fracture of coccyx with complete cauda equina lesion, |
| 318590010 | Closed fracture of coccyx with other cauda equina injury, |
| 318591014 | Closed fracture of coccyx with other spinal cord injury, |
| 318592019 | Closed fracture of coccyx with spinal cord lesion NOS, |
| 318593012 | Open fracture of coccyx with spinal cord lesion, |
| 318594018 | Open fracture of coccyx with unspecified spinal cord lesion, |
| 318595017 | Open fracture of coccyx with complete cauda equina lesion, |
| 318596016 | Open fracture of coccyx with other cauda equina injury, |
| 318597013 | Open fracture of coccyx with other spinal cord injury, |
| 318598015 | Open fracture of coccyx with spinal cord lesion NOS, |
| 318599011 | Closed fracture of spine with spinal cord lesion unspecified, |
| 318600014 | Open fracture of spine with spinal cord lesion unspecified, |
| 318601013 | Fracture of spine with spinal cord lesion NOS, |
| 318725011 | Multiple fractures of thoracic spine, |
| 318739011 | Closed fracture scapula, spine, |
| 318746019 | Open fracture scapula, spine, |
| 319203012 | Closed fracture spine, tibia, |
| 319215011 | Open fracture spine, tibia, |
| 325247012 | Fractures involving thorax with lower back and pelvis, |
| 325323015 | [X]Fracture of other specified cervical vertebra, |
| 391329016 | Closed fracture of atlas without spinal cord lesion, |
| 391330014 | Closed fracture of axis without spinal cord lesion, |
| 391337012 | Open fracture of atlas without spinal cord lesion, |
| 391340012 | Open fracture of axis without spinal cord lesion, |
| 394586014 | Other reduction of fracture of spine, |
| 402831015 | Fracture of vertebra without spinal cord lesion, |
| 402832010 | Fracture of spine without mention of spinal cord injury, |
| 402833017 | Closed fracture of cervical spine, |
| 402834011 | Closed fracture atlas, |
| 402835012 | Closed fracture axis, |
| 402836013 | Closed fracture of third cervical vertebra, |
| 402837016 | Closed fracture of fourth cervical vertebra, |
| 402838014 | Closed fracture of fifth cervical vertebra, |
| 402839018 | Closed fracture of sixth cervical vertebra, |
| 402840016 | Closed fracture of seventh cervical vertebra, |
| 402841017 | Open fracture of cervical spine, |
| 402844013 | Open fracture of third cervical vertebra, |
| 402845014 | Open fracture of fourth cervical vertebra, |
| 402846010 | Open fracture of fifth cervical vertebra, |
| 402847018 | Open fracture of sixth cervical vertebra, |
| 402848011 | Open fracture of seventh cervical vertebra, |
| 402849015 | Fracture of vertebra with spinal cord lesion, |
| 402850015 | Fracture of spine with spinal cord lesion, |
| 405143010 | Pathological fracture of thoracic vertebra, |
| 405144016 | Pathological fracture of lumbar vertebra, |
| 411017011 | Other reduction of fracture of spine and stabilisation, |
| 411019014 | Internal fixation of fracture of spine, |
| 451463012 | Pathological fracture of cervical vertebra, |
| 455444016 | Closed multiple fractures of thoracic spine, |
| 455445015 | Open multiple fracture of thoracic spine, |
| 456414012 | Late effect of fracture of cervical vertebra, |
| 456415013 | Late effect of fracture of thoracic vertebra, |
| 456416014 | Late effect of fracture of lumbar vertebra, |
| 1220723013 | Fracture of thoracic vertebra, |
| 1220724019 | Fracture of lumbar vertebra, |
| 1485121013 | H/O vertebral fracture, |
| 2674037014 | Balloon kyphoplasty of fracture of spine, |
| 570851000006111 | Cls spinal fracture wth anterior thoracic cord lesion,T1-6, |
| 12452461000006116 | Primary closed reduction spinal fracture and collar stabilisation, |
| 171431000006117 | Revision cls reduc spinal fracture+other external stabilistn, |
| 204961000006112 | Primary open reduc spinal fracture+internal fix+plate, |
| 43221000006113 | Opn spinal fracture with unspec thoracic cord lesion, T1-6, |
| 531621000006112 | C3 vertebra closed fracture without spinal cord lesion, |
| 4685081000006112 | Revision to skull traction stabilization of spinal fracture, |
| 6006171000006118 | Revision closed reduction of spinal fracture, |
| 159381000006114 | Rvsn open reduc spinal fracture+cast stabilisation, |
| 387541000006118 | [X]Fract of other and unspec parts of lumbar spine & pelvis, |
| 4684921000006113 | Primary bedrest stabilization of spinal fracture, |
| 562351000006114 | Closed fracture inv thorax wth low back and pelvis and limbs, |
| 159471000006115 | Rvsn open reduc spinal fracture+skull traction stabilisation, |
| 43151000006112 | Opn spinal fracture with complete thorac cord lesion, T7-12, |
| 4685151000006117 | Primary closed reduction cervical spine fracture and collar stabilization, |
| 4685311000006115 | Revision to closed reduction spinal fracture and cast stabilization, |
| 4685061000006119 | Revision to collar stabilization of cervical spine fracture, |
| 171411000006111 | Revision cls reduc spinal fracture+cast stabilisation, |
| 12729431000006111 | Fracture of spine without mention of spinal cord lesion NOS, |
| 4840571000006113 | Multiple fractures of lumbar spine and/or pelvis, |
| 159361000006116 | Rvsn open reduc spinal #+internal fix+segmental wire system, |
| 563691000006115 | Closed fracture of sacrum with unspec spinal cord lesion, |
| 43201000006115 | Opn spinal fracture with unspec cervical cord lesion, C1-4, |
| 6006191000006117 | Revision open reduction of spinal fracture, |
| 895351000006119 | #Sacrum/coccyx + cord lesion, |
| 4685131000006112 | Primary closed reduction cervical spine fracture and collar stabilisation, |
| 5461851000006118 | Open fracture of first cervical vertebra without spinal cord lesion, |
| 857661000006110 | Wedge compression # lumbar spine, |
| 570781000006113 | Cls spinal fracture with posterior cervcl cord lesion, C5-7, |
| 12221011000006118 | Open fracture tibial spine, |
| 4685331000006114 | Primary open reduction of cervical spine fracture and collar stabilisation, |
| 895281000006117 | #Thoracic spine-no cord lesion, |
| 4840551000006115 | Fracture of lumbar spine and/or pelvis, |
| 895331000006114 | #Thoracic spine + cord lesion, |
| 570741000006119 | Cls spinal fracture with complete cervcl cord lesion, C1-4, |
| 7365351000006118 | Infracoccygeal uteropexy, |
| 570671000006117 | Cls spinal fracture with anterior cervcl cord lesion, C1-4, |
| 1682801000000118 | Removal of fracture fixation device from spine, |
| 2526081000006114 | Fracture of vertebral column with spinal cord injury, |
| 44401000006117 | Opn spinal # with incomplete thoracic cord lesion, T7-12 NOS, |
| 531571000006114 | C1 vertebra closed fracture - no spinal cord lesion, |
| 3316961000006117 | Fracture of spine, |
| 4840621000006111 | Closed spinal fracture with central cervical cord lesion, C1-4, |
| 12220961000006113 | Closed fracture intercondylar spine of tibia, |
| 4685171000006110 | Primary closed reduction spinal fracture and bedrest stabilization, |
| 204981000006119 | Primary open reduc spinal fracture+internal fix+wire, |
| 5560691000006114 | Fracture of lumbar spine with cord lesion, |
| 531641000006117 | C4 vertebra closed fracture without spinal cord lesion, |
| 159451000006113 | Rvsn open reduc spinal fracture+other external stabilisation, |
| 531711000006112 | C7 vertebra open fracture without spinal cord lesion, |
| 44381000006117 | Opn spinal # with incomplete cervical cord lesion, C5-7 NOS, |
| 570811000006110 | Cls spinal fracture with unspec cervical cord lesion, C1-4, |
| 503551000006112 | Barr skull traction for fracture of spine, |
| 4685041000006118 | Revision to collar stabilisation of cervical spine fracture, |
| 793671000006119 | Fracture of transverse process of spine + spinal cord lesion, |
| 44451000006118 | Opn spinal fracture with complete cervcl cord lesion, C1-4, |
| 895301000006118 | #Sacrum/coccyx-no cord lesion, |
| 4685011000006117 | Primary external fixation stabilization of spinal fracture, |
| 531691000006114 | C6 vertebra open fracture without spinal cord lesion, |
| 990941000006118 | #Spine NOS - no cord lesion, |
| 210121000006110 | Primary cls reduction spinal fracture+cast stabilisation, |
| 44441000006115 | Opn spinal fracture with anterior thorac cord lesion, T7-12, |
| 210111000006119 | Primary cls reduction spinal fracture+bedrest stabilisation, |
| 3316991000006113 | Fracture of vertebra, |
| 12451691000006118 | Revision to open reduction spinal fracture and collar stabilisation, |
| 375761000000118 | Posterior decompression of fracture of spine NEC, |
| 12451851000006113 | Revision to closed reduction spinal fracture and collar stabilisation, |
| 570701000006116 | Cls spinal fracture with central cervical cord lesion, C1-4, |
| 4841521000006118 | Closed fracture dislocation of sacroiliac joint, |
| 570721000006114 | Cls spinal fracture with central thoracic cord lesion, T1-6, |
| 44431000006113 | Opn spinal fracture with anterior thorac cord lesion, T1-6, |
| 2649011000006118 | Sequelae of fracture of spine, |
| 761191000006114 | Fixation of fracture of spine, |
| 44391000006119 | Opn spinal # with incomplete thoracic cord lesion, T1-6 NOS, |
| 895321000006111 | #Cervical spine + cord lesion, |
| 4685291000006119 | Revision to closed reduction spinal fracture and skull traction stabilization, |
| 531681000006111 | C6 vertebra closed fracture without spinal cord lesion, |
| 899151000006119 | Spinal cord lesion - no #, |
| 4685391000006113 | Primary open reduction spinal fracture and skull traction stabilization, |
| 210131000006113 | Primary cls reduction spinal fracture+collar stabilisation, |
| 531661000006118 | C5 vertebra closed fracture without spinal cord lesion, |
| 44411000006119 | Opn spinal fracture with anterior cervcl cord lesion, C1-4, |
| 4685271000006115 | Revision to closed reduction spinal fracture and bedrest stabilization, |
| 2649001000006116 | Late effect of fracture of spine AND/OR trunk without spinal cord lesion, |
| 5560671000006113 | Fracture of cervical spine with cord lesion, |
| 4685431000006119 | Revision to open reduction of cervical spine fracture and collar stabilisation, |
| 5509791000006111 | Fracture of spine without spinal cord injury, |
| 3316971000006112 | Spinal fracture, |
| 531671000006113 | C5 vertebra open fracture without spinal cord lesion, |
| 4685411000006113 | Primary open reduction spinal fracture and cast stabilization, |
| 204941000006113 | Primary open reduc spinal #+intern fix+segmental wire system, |
| 602751000006117 | Crutchfield skull traction for fracture of spine, |
| 570831000006116 | Cls spinal fracture with unspec thoracic cord lesion, T7-12, |
| 750591000006119 | Late effect of fracture of spine/trunk without cord lesion, |
| 3316981000006110 | Fracture of spinal vertebra, |
| 2812621000006110 | Open reduction of spinal fracture, |
| 563961000006116 | Closed fracture of thoracic spine with cord lesion NOS, |
| 4684931000006111 | Primary collar stabilisation of cervical spine fracture, |
| 12484081000006118 | Closed fracture spine, tibia, |
| 570841000006114 | Cls spinal fracture with unspec thoracic cord lesion,T1-6, |
| 570691000006116 | Cls spinal fracture with anterior thorac cord lesion, T7-12, |
| 259481000006119 | Open fracture inv thorax wth low back and pelvis and limbs, |
| 895311000006115 | #Spine NOS - no cord lesion, |
| 204951000006110 | Primary open reduc spinal #+internal fix+internal fixator, |
| 5509811000006110 | Closed fracture of first cervical vertebra, |
| 570711000006118 | Cls spinal fracture with central cervical cord lesion, C5-7, |
| 44421000006110 | Opn spinal fracture with anterior cervcl cord lesion, C5-7, |
| 205441000006119 | Primary opn reduc spinal fracture+other external stabilisatn, |
| 259761000006117 | Open fracture dislocation of sacro-iliac joint, |
| 531701000006114 | C7 vertebra closed fracture without spinal cord lesion, |
| 857671000006115 | Wedge compression # of dorsal spine, |
| 159461000006110 | Rvsn open reduc spinal fracture+other internal fix, |
| 4841671000006118 | Multiple fracture of thoracic vertebrae, |
| 895291000006119 | #Lumbar spine - no cord lesion, |
| 43141000006110 | Opn spinal fracture with complete thorac cord lesion, T1-6, |
| 265391000006117 | Op spinal fracture with central thoracic cord lesion, T7-12, |
| 5509831000006116 | Closed fracture of second cervical vertebra, |
| 570861000006113 | Cls spinal fracture wth complete thoracic cord lesion,T1-6, |
| 895361000006117 | #Spine NOS + cord lesion, |
| 12220991000006117 | Open fracture intercondylar spine of tibia, |
| 6006161000006113 | Primary closed reduction of spinal fracture, |
| 531631000006110 | C3 vertebra open fracture without spinal cord lesion, |
| 43211000006117 | Opn spinal fracture with unspec cervical cord lesion, C5-7, |
| 12452471000006111 | Primary collar stabilisation of spinal fracture, |
| 171441000006110 | Revision cls reduc spinal fracture+skull traction stabilistn, |
| 159371000006111 | Rvsn open reduc spinal fracture+bedrest stabilisation, |
| 570771000006110 | Cls spinal fracture with posterior cervcl cord lesion, C1-4, |
| 204971000006117 | Primary open reduc spinal fracture+internal fix+rod system, |
| 159441000006111 | Rvsn open reduc spinal fracture+internal fix+wire, |
| 570681000006119 | Cls spinal fracture with anterior cervcl cord lesion, C5-7, |
| 370001000000111 | Vertebroplasty of fracture of spine, |
| 6518851000006116 | History of vertebral fracture, |
| 205451000006117 | Primary opn reduc spinal fracture+skull traction stabilisatn, |
| 204991000006116 | Primary open reduc spinal fracture+other internal fix, |
| 562841000006118 | Closed fracture of coccyx with unspec spinal cord lesion, |
| 159391000006112 | Rvsn open reduc spinal fracture+collar stabilisation, |
| 43231000006111 | Opn spinal fracture with unspec thoracic cord lesion, T7-12, |
| 698931000006114 | Manipulative reduction of fracture of spine, |
| 43161000006114 | Opn spinal fracture with posterior cervcl cord lesion, C1-4, |
| 570751000006117 | Cls spinal fracture with complete cervcl cord lesion, C5-7, |
| 4685231000006118 | Revision to closed reduction of cervical spine fracture and collar stabilisation, |
| 531651000006115 | C4 vertebra open fracture without spinal cord lesion, |
| 570661000006112 | Cls spinal # with incomplete thoracid cord lesion, T7-12 NOS, |
| 205471000006110 | Primary opn reduction spinal fracture+bedrest stabilisation, |
| 4684951000006116 | Primary collar stabilization of cervical spine fracture, |
| 570821000006119 | Cls spinal fracture with unspec cervical cord lesion, C5-7, |
| 2687681000006118 | Closed fracture of axis without spinal cord injury, |
| 793681000006116 | Fracture of transverse process spine - no spinal cord lesion, |
| 4685031000006111 | Revision to bedrest stabilization of spinal fracture, |
| 12728571000006118 | Fracture of spine with spinal cord lesion NOS, |
| 761201000006112 | Fixation of fracture of spine and skull traction HFQ, |
| 570731000006112 | Cls spinal fracture with central thoracid cord lesion, T7-12, |
| 5560681000006111 | Fracture of thoracic spine with cord lesion, |
| 210141000006115 | Primary cls reduction spinal fracture+external fixation, |
| 4685251000006113 | Revision to closed reduction of cervical spine fracture and collar stabilization, |
| 4685121000006114 | Revision to external fixation stabilization of spinal fracture, |
| 4378891000006113 | Fracture of lumbar spine, |
| 570791000006111 | Cls spinal fracture with posterior thorac cord lesion, T1-6, |
| 895271000006115 | #Cervical spine-no cord lesion, |
| 12220971000006118 | Closed fracture of tibial spine, |
| 4841581000006119 | Open fracture dislocation of sacroiliac joint, |
| 171421000006115 | Revision cls reduc spinal fracture+external fixation, |
| 4684881000006117 | Manipulation of spinal fracture, |
| 895341000006116 | #Lumbar spine + cord lesion, |
| 562751000006110 | Closed fracture of cervical spine with cord lesion NOS, |
| 570631000006115 | Cls spinal # with incomplete cervical cord lesion, C1-4 NOS, |
| 4685451000006114 | Revision to open reduction of cervical spine fracture and collar stabilization, |
| 4685371000006112 | Primary open reduction spinal fracture and bedrest stabilization, |
| 210051000006117 | Primary cls reduc spinal fracture+skull traction stabilisatn, |
| 4684991000006110 | Primary cast stabilization of spinal fracture, |
| 44461000006116 | Opn spinal fracture with complete cervcl cord lesion, C5-7, |
| 40891000006118 | Osteoporosis + pathological fracture thoracic vertebrae, |
| 4685471000006116 | Revision to open reduction spinal fracture and bedrest stabilization, |
| 159401000006114 | Rvsn open reduc spinal fracture+external fix, |
| 570641000006113 | Cls spinal # with incomplete cervical cord lesion, C5-7 NOS, |
| 12732571000006118 | Other specified decompression of fracture of spine, |
| 44371000006115 | Opn spinal # with incomplete cervical cord lesion, C1-4 NOS, |
| 4685101000006116 | Revision to cast stabilization of spinal fracture, |
| 12053961000006111 | Open fracture of coccyx, |
| 210061000006115 | Primary cls reduct spinal fracture+oth external stabilisatn, |
| 570801000006112 | Cls spinal fracture with posterior thorac cord lesion, T7-12, |
| 4685511000006114 | Revision to open reduction spinal fracture and cast stabilization, |
| 159411000006112 | Rvsn open reduc spinal fracture+internal fix+internl fixator, |
| 4378851000006119 | Fracture of cervical vertebra, |
| 51781000006118 | Open spinal fracture with incomplete lumbar cord lesion NOS, |
| 570651000006110 | Cls spinal # with incomplete thoracic cord lesion, T1-6 NOS, |
| 43191000006118 | Opn spinal fracture with posterior thorac cord lesion, T7-12, |
| 205481000006113 | Primary opn reduction spinal fracture+cast stabilisation, |
| 4684971000006114 | Primary skull traction stabilization of spinal fracture, |
| 40871000006119 | Osteoporosis + pathological fracture cervical vertebrae, |
| 171491000006118 | Revision cls reduction spinal fracture+collar stabilisation, |
| 4685491000006115 | Revision to open reduction spinal fracture and skull traction stabilization, |
| 4685191000006111 | Primary closed reduction spinal fracture and skull traction stabilization, |
| 4378871000006112 | Fracture of thoracic spine, |
| 159421000006116 | Rvsn open reduc spinal fracture+internal fix+plate, |
| 4816081000006112 | Stress fracture of vertebra, |
| 4685351000006119 | Primary open reduction of cervical spine fracture and collar stabilization, |
| 171401000006113 | Revision cls reduc spinal fracture+bedrest stabilisation, |
| 531581000006112 | C1 vertebra open fracture without spinal cord lesion, |
| 5461881000006114 | Open fracture of second cervical vertebra without spinal cord lesion, |
| 12452431000006113 | Primary open reduction spinal fracture and collar stabilisation, |
| 531601000006119 | C2 vertebra closed fracture without spinal cord lesion, |
| 43181000006116 | Opn spinal fracture with posterior thorac cord lesion, T1-6, |
| 3317001000006117 | Fractured spine, |
| 159431000006118 | Rvsn open reduc spinal fracture+internal fix+rod system, |
| 12453181000006110 | Open fracture spine, tibia, |
| 166841000006113 | Revision to external fixation stabilisation spinal fracture, |
| 205491000006111 | Primary opn reduction spinal fracture+collar stabilisation, |
| 4685211000006112 | Primary closed reduction spinal fracture and cast stabilization, |
| 43171000006119 | Opn spinal fracture with posterior cervcl cord lesion, C5-7, |
| 531611000006116 | C2 vertebra open fracture without spinal cord lesion, |
| 570761000006115 | Cls spinal fracture with complete thorac cord lesion, T7-12, |
| 561821000006117 | Closed fracture dislocation of sacro-iliac joint, |
| 40881000006116 | Osteoporosis + pathological fracture lumbar vertebrae, |
| 43041000006111 | Open reduction of fracture of spine & excis facet of spine, |
| 6006181000006115 | Primary open reduction of spinal fracture, |
| 12451801000006114 | Revision to collar stabilisation of spinal fracture |

**Table S1: The adjusted model of patients diagnosed with OSA before the diagnosis of T2D (exposed) and patients without OSA (unexposed).**

Continuous data are presented as median (IQR) or mean (SD). Categorical variables are presented as n (%).

| Hazard Ratio | HR (95% CI) | P value |
| --- | --- | --- |
| Unadjusted HR | 1.13 (1.02 - 1.26) | 0.02 |
| Adjusted HR | 1.12 (1.00 - 1.25) | 0.04 |
| Age | 1.04 (1.04 - 1.05) | <0.00 |
| Sex: Female | 2.12 (1.9 - 2.35) | <0.00 |
| Ethnicity:  Black  South Asian  Mixed race  Others  Missing | 0.32 (0.21 - 0.47)  0.63 (0.49 - 0.80)  0.40 (0.14 - 1.06)  0.86 (0.50 – 1.49)  1.08 (0.92 -1.25) | <0.00  <0.00  0.06  0.60  0.32 |
| Charlson Comorbidity Index | 1.05 (1.02 – 1.07) | <0.00 |
| BMI:  25–30 kg/m2  > 30 kg/m2  Missing | - 1. (0.75 - 1.36)   0.84 (0.63 - 1.11)  0.90 (0.64 – 1.25) | 0.93  0.23  0.55 |
| Smoking status:  Ex-Smoker  Current Smoker  Missing | 0.97 (0.85 - 1.10)  1.11 (0.98 - 1.27)  1.29 (1.00 - 1.68) | 0.76  0.09  1.00 |
| Alcohol | 1.67 (1.39 - 2.01) | <0.00 |
| Townsend deprivation quintile  2  3  4  5  Missing | 1.04 (0.88 - 1.22)  1.17 (1.00 - 1.37)  1.18 (1.01 - 1.38)  1.22 (1.04 - 1.43)  0.89 (0.49 - 131) | 0.59  0.05  0.04  0.01  0.38 |
| eGFR category  60–89 (stage 2)  30–59 (stage 3)  <30 (stage4)  Missing | 0.74 (0.66 - 0.84)  0.71 (0.59 - 0.85)  1.23 (0.70 - 2.17)  0.67 (0.56 - 0.81) | <0.00  <0.00  0.46  <0.00 |
| HbA1c:  6.5 -7.5 %  7.5 -8.5 %  ≥ 8.5 %  Missing | 1.07 (0.88 - 1.30)  0.99 (0.74 - 1.31)  0.99 (0.79 - 1.26)  0.92 (0.79 -1.06) | 0.46  0.96  0.98  0.27 |
| Hypoglycaemia | 2.47 (1.51 - 4.03) | <0.00 |
| Hyperthyroidism | 1.18 (0.56 - 2.549) | 0.66 |
| Baseline drug use  Glucose-lowering drugs  Systemic steroids  Bisphosphonate  Insulin  Calcium supplements  Vitamin D | 1.09 (0.98 - 1.21)  1.15 (1.04 - 1.28)  1.42 (1.08 - 1.88)  1.59 (1.14 - 1.21)  1.13 (0.83 – 1.54)  0.85 (0.65 – 1.11) | 0.09  0.01  0.01  0.01  0.43  0.24 |

Abbreviations: IQR, intra-quantile range; CI, confidence interval; HR, hazard rate ratio. P values derived from Cox regression.

Adjusted for age, sex, body mass index, smoking, Townsend, ethnicity, insulin use, glucose-lowering agents, steroid use, bisphosphonate use, vitamin D, calcium supplement, alcohol, eGFR, Hypoglycaemia, HbA1c, Carlson Comorbidity Index, and hyperthyroidism

**Table S2: The adjusted model of patients diagnosed with OSA after the diagnosis of T2D (exposed) and patients without OSA (unexposed)**

Continuous data are presented as median (IQR) or mean (SD). Categorical variables are presented as n (%).

| Hazard Ratio | HR (95% CI) | P value |
| --- | --- | --- |
| Unadjusted HR | 1.21 (1.10 - 1.32) | <0.001 |
| Adjusted HR | 1.15 (1.05 - 1.26) | 0.002 |
| Age | 1.04 (1.03 - 1.05) | <0.001 |
| Sex: Female | 2.03 (1.86 – 2.20) | <0.001 |
| Ethnicity:  Black  South Asian  Mixed race  Others  Missing | 0.39 (0.30 - 0.51)  0.69 (0.58 - 0.82)  0.83 (0.47 - 1.47)  0.52 (0.29 - 0.92)  1.17 (1.03 - 1.33) | <0.001  <0.001  0.53  0.03  0.02 |
| Charlson Comorbidity Index | 1.06 (1.04 - 1.08) | <0.001 |
| BMI:  25–30 kg/m^2^  > 30 kg/m^2^  Missing | 0.82 (0.64 - 1.06)  0.69 (0.55 - 0.88)  0.79 (0.56 - 1.09) | 0.13  0.00  0.16 |
| Smoking status:  Ex-Smoker  Current Smoker  Missing | 1.08 (0.97 - 1.21)  1.18 (1.05 - 1.33)  1.11 (0.80 - 1.55) | 0.12  0.01  0.50 |
| Alcohol | 1.52 (1.30 -1.79) | <0.001 |
| Townsend deprivation quintile  2  3  4  5  Missing | 1.09(0.95 - 1.25)  1.07 (0.93 - 1.22)  1.10 (0.96 -1.26)  1.13 (0.99 - 1.29)  1.04 (0.72 - 1.50 | 0.17  0.32  0.13  0.05  0.82 |
| eGFR category  60–89 (stage 2)  30–59 (stage 3)  <30 (stage4)  Missing | 0.89 (0.80 - 0.98)  0.90 (0.79 - 1.04)  1.36 (1.05 -1.77)  1.13 (0.95 - 1.34) | 0.03  0.17  1.02  1.14 |
| HbA1c:  6.5 -7.5 %  7.5 -8.5 %  ≥ 8.5 %  Missing | 0.95 (0.84 - 1.07)  1.10 (0.95 - 1.26)  1.05 (0.91 - 1.22)  0.98 (0.87 - 1.10) | 0.46  0.18  0.43  0.74 |
| Hypoglycaemia | 1.04 (0.84 - 1.28) | 0.70 |
| Diabetes Duration | 1.01 (1.00 - 1.01) | 0.02 |
| Hyperthyroidism | 0.85 (0.50 -1.41) | 0.53 |
| Baseline drug use  Glucose-lowering drugs  Systemic steroids  Bisphosphonate  Insulin  Calcium supplements  Vitamin D | 1.08 (0.97 - 1.21)  1.16 (1.06 - 1.26)  1.46 (1.21 - 1.77)  1.20 (1.07 - 1.34)  1.20 (0.97 - 1.47)  1.03 (0.85 - 1.24) | 0.14  0.00  <0.001  0.00  0.08  0.74 |

Abbreviations: IQR, intra quantile range; CI, confidence interval; HR, hazard rate ratio. P values derived from Cox regression.

Adjusted for age, sex, body mass index, smoking, Townsend, ethnicity, duration of diabetes, insulin use, glucose-lowering agents, steroid use, bisphosphonates use, vitamin D, calcium supplement, alcohol, eGFR, Hypoglycaemia, HbA1c, Carlson Comorbidity Index, and hyperthyroidism
